# Education, Employment, and Financial Outcomes in Adolescent and Young Adult Cancer Survivors—A Systematic Review

**DOI:** 10.3390/curroncol30100631

**Published:** 2023-09-25

**Authors:** Aurelia Altherr, Céline Bolliger, Michaela Kaufmann, Daniela Dyntar, Katrin Scheinemann, Gisela Michel, Luzius Mader, Katharina Roser

**Affiliations:** 1 Faculty of Health Sciences and Medicine, University of Lucerne, Alpenquai 4, 6005 Lucerne, Switzerland; aurelia.altherr@gmail.com (A.A.); celine.bolliger@unilu.ch (C.B.); daniela.dyntar@unibe.ch (D.D.); katrin.scheinemann@kispisg.ch (K.S.); gisela.michel@unilu.ch (G.M.); 2 Cancer Registry of Central Switzerland, 6000 Lucerne, Switzerland; 3 Division of Hematology & Oncology, Children’s Hospital of Eastern Switzerland, 9006 St. Gallen, Switzerland; 4 Department of Pediatrics, McMaster Children’s Hospital and McMaster University, Hamilton, ON L8N 3Z5, Canada; 5 Institute of Social and Preventive Medicine, University of Bern, 3012 Bern, Switzerland; luzius.mader@unibe.ch; 6 Cancer Registry Bern-Solothurn, University of Bern, 3008 Bern, Switzerland

**Keywords:** adolescent and young adult, cancer, survivors, education, employment, financial outcomes, psychosocial health

## Abstract

Adolescents and young adults (AYAs) with cancer face unique challenges. We aimed to describe (i) education, employment, and financial outcomes and (ii) determinants for adverse outcomes in AYA cancer survivors. We performed a systematic literature search. We included original research articles on AYA (15–39 years of age) cancer survivors (≥2 years after diagnosis) and our outcomes of interest. We narratively synthesized the results of the included articles. We included 35 articles (24 quantitative and 11 qualitative studies). Patients in education had to interrupt their education during cancer treatment, and re-entry after treatment was challenging. After treatment, most survivors were employed but started their employment at an older age than the general population. Overall, no disadvantages in income were found. Survivors reported more absent workdays than comparisons. We identified chemotherapy, radiotherapy, late effects or health problems, female sex, migration background, and lower education associated with adverse outcomes. Although most AYA cancer survivors were able to re-enter education and employment, they reported difficulties with re-entry and delays in their employment pathway. To facilitate successful re-entry, age-tailored support services should be developed and implemented.

## 1. Introduction

AYAs are diagnosed with cancer during a unique and challenging period of their life [1,2]. The transitional time between childhood and adulthood is characterized by psychosocial milestones related to completing education, starting their employment pathway, and gaining social and financial independence from parents [1,3,4,5,6,7,8]. The cancer diagnosis may interfere with these psychosocial achievements. It has been shown that psychosocial problems after cancer are more prevalent in AYAs than in older adults [9]. This indicates that cancer might be especially disruptive in AYAs and emphasizes the importance of psychosocial health in AYA cancer survivors.

Cancer in young people is different from cancer in children or cancer in older adults: The epidemiology, the biology of the tumors, and the psychosocial needs of AYA cancer survivors and late outcomes after the cure of the cancer are unique in this specific age group [10,11,12,13,14]. In Europe, about 112′000 AYAs were diagnosed with cancer in 2020 [15]. Survival nowadays exceeds 80% in Europe [16].

The majority of AYA cancer survivors returned to school or work after the end of treatment [17]. However, many AYA cancer survivors reported that cancer had a negative impact on their plans for work or school [17] and that returning to work was challenging [18]. Regarding survivors’ educational achievements, some studies indicate different educational pathways for survivors compared to the general population [19,20]. Other studies did not find any differences in educational attainment between survivors and comparisons [21]. However, survivors reported disruptions in their education due to the cancer diagnosis [21]. Regarding employment, some studies did not report an increased risk of unemployment in survivors [19,20]. They started being engaged in paid employment at an older age compared to the general population [20]. In other studies, survivors were less likely to be employed compared to the general population [21,22], and this difference was especially pronounced for health-related unemployment [21].

Cancer and its treatment and disruptions or delays in employment might lead to financial hardship. Different pathways have been suggested for this adverse outcome. Many survivors experience chronic conditions, which are associated with significant increases in medical expenditures and health care use [23]. Furthermore, different educational pathways and a higher risk of unemployment might also increase financial hardship [24,25,26].

A comprehensive overview of education, employment, and financial outcomes in survivors of AYA cancer is lacking. This systematic review aimed to describe (i) education, employment, and financial outcomes and (ii) determinants for adverse educational, employment, and financial outcomes in AYA cancer survivors.

## 2. Methods

This systematic review was registered in PROSPERO (number: CRD42021262353) and complies with the PRISMA statement regarding reporting systematic reviews and meta-analyses [27].

### 2.1. Search Strategy

The literature search was conducted in August 2020 and updated on 15 February 2022. We searched the databases PubMed, Scopus, and PsychINFO. Included publications were hand-searched for additional references. No restrictions on geographical region or publication language were applied. The search was restricted to studies on humans that were published up to 15 February 2022. The search terms included four blocks with search terms referring to the outcomes (education, work, financial outcomes), adolescent and young adult, cancer, and survivorship (Tables S3 and S4 in Supplementary Material).

### 2.2. Study Selection

The study selection consisted of two steps: title and abstract screening and full text screening.

To select eligible articles, the following inclusion criteria were hierarchically applied: peer-reviewed original research, a sample size of at least 20 for quantitative studies (no sample size restrictions for qualitative and mixed methods studies), study participants having been diagnosed with cancer, AYA cancer (i.e., at least 75% of participants in the age range of 15–39 years at diagnosis), survivors (i.e., at least 75% of participants at least two years after diagnosis), and one of the three outcomes of interest being the primary outcome presented in the article (education, employment, financial outcomes). Review articles, editorials, commentaries, and conference abstracts were excluded. During the full-text screening, articles from which no full text could be obtained were excluded.

We included quantitative, qualitative, and mixed methods studies and any study designs. Studies with and without comparisons (e.g., general population, siblings) were included. Two reviewers each independently assessed eligibility by first screening titles and abstracts followed by the full texts of the remaining articles (involved authors: A.A., C.B., M.K., K.R.). Discrepancies between reviewers were resolved by discussion and consensus or by consulting a third reviewer (L.M.). Reference lists of relevant review articles were screened for potentially eligible articles.

### 2.3. Data Extraction

The first author, publication year, country, study design, data source, data collection method, sample size, response rate, and population characteristics, including gender, age at time of study, age at diagnosis, time since diagnosis, cancer types, and education, employment, and financial information (which were mentioned additionally to the primary outcomes of the articles), were extracted. If a comparison group was available, the provided information was extracted as well (Table 1 and Table S1 in Supplementary Material for quantitative studies and Table 2 and Table S2 in Supplementary Material for qualitative studies).

### 2.4. Quality Assessment

The quality of each study was independently assessed by two reviewers each using the JBI critical appraisal tool [28] (involved authors: A.A., M.K., K.R.). Discrepancies between reviewers were resolved by discussion and consensus. Inter-rater reliability, assessed by Kendall’s tau, was tau = 0.74 for quantitative studies and tau = 0.71 for qualitative studies. The JBI critical appraisal tool was designed to assess methodological validity and determine the extent to which a study considered possible biases in its design, conduct, and analysis. It is suitable for cross-sectional, cohort, and qualitative studies, which are common in this research area [27]. To assess study quality, 8 questions were asked for cross-sectional studies and 10 questions for qualitative studies. These items could be answered with “yes,” “no,” “unclear,” or “not applicable.” To enable a comparable assessment across cross-sectional studies and qualitative studies, the total number of questions answered with “yes” was summed up, and the percentage of “yes” answers was calculated. For cross-sectional studies, a maximum of 8 “yes” and for qualitative studies, a maximum of 10 “yes” answers could be reached (Tables S5 and S6 in Supplementary Material).

### 2.5. Data Synthesis

Outcomes related to the psychosocial situation of AYA cancer survivors were narratively synthesized. A priori, we did not consider a meta-analytic approach because of the expected heterogeneity in study design, study period, outcome definition across studies, and differences in educational, labor, and financial contexts across geographic regions. The narrative synthesis focused on the educational, employment, and financial outcomes and the determinants for adverse educational, employment, and financial outcomes. Further, the quality of the included studies was evaluated to determine how it may have influenced the synthesis.

**Table 1:** Characteristics of included quantitative studies.

First Author, Publication Year	Country	Study Design	Sample Size	Response Rate	Gender: Percentage Male	Age at Time of Study	Age at Diagnosis	Time Since Diagnosis	Cancer Types	Comparisons	Study Quality
Abdelhadi et al., 2021 [23]	USA	Retrospective cohort study	*n* = 2326	MEPS (2011–2016): 53.5–59.3% for the different years	AYA cancer survivors with chronic conditions: 23.90% male, AYA cancer survivors without chronic conditions: 21.85% male	(Weighted proportions) AYA cancer survivors with chronic conditions: 18–29 years old: 6.14%, 30–39 years old: 15.52%, 40–49 years old: 24.36%, 50–64 years old: 36.10%, ≥65 years old: 17.88% AYA cancer survivors without chronic conditions: 18–29 years old: 18.14%, 30–39 years old: 37.52%, 40–49 years old: 27.82%, 50–64 years old: 13.90%, ≥65 years old: 2.70%	range: 15–39 years	AYA cancer survivors with chronic conditions: 0–4 years: 10.86%, 5–9 years: 12.73%, 10–19 years: 26.31%, ≥20 years: 50.09% AYA cancer survivors without chronic conditions: 0–4 years: 31.85%, 5–9 years: 22.96%, 10–19 years: 29.43%, ≥20 years: 15.76%	(Weighted proportions) AYA cancer survivors with chronic conditions: bladder: 0.70%, brain: 1.69%, breast: 12.57%, cervix: 32.90%, colon: 2.94%, leukemia: 1.72%, lung: 2.07%, lymphoma: 4.42%, melanoma: 9.26%, other: 28.26%, prostate: 1.70%, throat: n/a, thyroid: 3.90% AYA cancer survivors without chronic conditions: bladder: n/a, brain: n/a, breast: 11.15%, cervix: 21.86%, colon: 1.76%, leukemia: 1.52%, lung: n/a, lymphoma: 5.45%, melanoma: 10.94%, other: 26.55%, prostate: n/a, throat: n/a, thyroid: 8.50%	None	88%
Abdelhadi et al., 2022 [29]	USA	Retrospective cohort study	*n* = 2081 (*n* = 1757 for matched analyses)	MEPS (2011–2016): 53.5–59.3% for the different years	20.0% male	18–29 years old: 10.2%, 30–39 years old: 22.9%, 40–49 years old: 27.3%, 50–64 years old: 26.6%, ≥65 years old: 13.0%	Range: 15–39 years	Not reported	Not reported	Adults without cancer history (*n* = 5227)	88%
Bhatt et al., 2021 [30]	USA	Retrospective cohort study	*n* = 1365	Not applicable	56% male	Not reported	Mean age at treatment = 30.8 years old, range: 18–39 years old, 18–24 years old: 19%, 25–29 years old: 26%, 30–34 years old: 27%, 35–39 years old: 28%	Median time since treatment = 60.6 months, range: 12–121 months	Leukemia: 68%, lymphoma: 11%, other malignant diseases: 10%, non-malignant disorders: 11%	None	100%
Dahl et al., 2019 [31]	Norway	Cross-sectional study	*n* = 1189	42%	27% male	Mean (SD) = 49.7 (7.8), median = 49 years, range: 27–65 years old	Mean (SD) = 33.0 (5.3), median = 35 years old, range: 19–39 years old	Median = 16 years, range: 6–31 years	Breast: 41%, colorectal: 12%, lymphoma: 19%, leukemia: 11%, melanoma: 17%	None	100%
Dieluweit et al., 2011 [20]	Germany	Cross-sectional study	*n* = 820	43.70%	49% male	Mean (SD) = 29.9 (6) years old	Mean (SD) = 15.8 (0.9) years old, range: 15–18 years old	Mean (SD) = 13.7 (6) years	Lymphoma: 30.5%, malignant bone tumor: 21.2%, leukemia: 19.3%, CNS tumors: 9.5%, soft tissue and other extraosseous sarcomas: 9.2%, germ cell tumors: 6.6%, other malignant epithelial neoplasms and malignant melanomas: 2.4%, renal tumors: 0.9%, neuroblastoma: 0.5%	Age-matched sample from the general population (German Socio- Economic Panel, *n* = 820)	100%
Ekwueme et al., 2016 [32]	USA	Cross-sectional study	*n* = 244	Not reported	All female	Mean (SD) = 39.42 (5.29) years old	Mean (SD) = 34.42 (6.95) years old, range: 18–44 years old	<2 years: 30.74%, 2–4 years: 28.69%, 5–10 years: 29.1%, ≥11 years: 11.48%	All breast	Women aged 18–44 without breast cancer (*n* = 82694), women aged 45–64 at diagnosis with breast cancer (*n* = 1508), women aged 45–64 without breast cancer (*n* = 52,586)	88%
Ghaderi et al., 2013 [33]	Norway	Retrospective cohort study	*n* = 2561	Not applicable	55.4% male (childhood and AYA cancer survivors)	Not reported	15–19 years old: 1019, 20–24 years old: 1542	Survivors were followed for mean = 13.2 years beginning 5 years after diagnosis (range: 0–39.3 years) (childhood and AYA cancer survivors)	Brain/CNS tumors: 18.2%, testis: 15.4%, lymphatic system: 14.4%, hematopoietic system: 12.9%, melanoma: 10.6%, other: 7.4%, thyroid gland and other endocrine glands: 7.3%, bone and connective tissue: 5.6%, kidney: 2.7%, eye: 2.2%, ovary: 2%, cervix uteri: 1.2% (childhood and AYA cancer survivors)	Childhood cancer survivors (0–14 years of age at diagnosis; *n* = 1470)	100%
Guy et al., 2014 [34]	USA	Retrospective cohort study	*n* = 1464	MEPS (2008–2011): 53.5–59.3%	22.2% male	18–29 years old: 11%, 30–39 years old: 21%, 40–49 years old: 26.7%, 50–64 years old: 29.3%, ≥65 years old: 12%	range: 15–39 years	0–9 years: 30.5%, 10–19 years: 27.7%, ≥20 years: 41.9%	Not reported	Adults without cancer in the pooled sample of 2008–2011 MEPS data (*n* = 86,865)	88%
Hamzah et al., 2021 [35]	Malaysia	Cross-sectional study	*n* = 400	Not reported	43.3% male	Mean (SD) = 29.1 (7.16) years old, range: 18–40, 18–20 years old: 12.5%, 21–25 years old: 27%, 26–30 years old: 17.8%, 31–35 years old: 12.8%, 36–40 years old: 30%	Not reported	>5 years	Leukemia: 32.25%, Hodgkin lymphoma: 10.0%, ovarian: 8.0%, ependymoma: 7.25%, breast: 6.25%, Wilms’ tumor: 5.75%, Ewing’s sarcoma: 5.75%, testicular: 3.5%, medulloblastoma: 3.5%, brain tumor: 3.25%, yolk sac tumor: 3%, liver cancer: 2.75%, papillary thyroid: 1.5%, nasopharyngeal cancer: 1.5%, neuroblastoma: 1.5%, intestinal: 1.25%, lung: 1%, germinoma: 1%, embryonal rhabdomyosarcoma: 1%	None	63%
Ketterl et al., 2019 [24]	USA	Cross-sectional study	*n* = 872	67%	27.2% male	Not reported	Females: mean (SD) = 32.3 (5.62) years old, males: mean (SD) = 29.8 (6.09) years old	Females: mean (SD) = 3.53 (1.49) years, males: mean (SD) = 3.40 (1.29) years	Breast: 27.6%, leukemia and lymphoma: 18.7%, endocrine system: 14.7%, skin: 9.3%, genital system: 10.9%, brain and other CNS tumors: 4.7%, bones and soft tissue: 4.1%, digestive system: 4.0%, oral cavity and pharynx: 2.9%, urinary system: 1.6%, others: 1.5%	None	100%
Landwehr et al., 2016 [36]	USA	Retrospective cohort study	*n* = 334	33.60%	20.4% male	Age at time of application submission: mean = 29.3 years old, median = 30.0 years old, 95% CI: 28.7–29.8, SD = 4.4 years old, range: 19–39 years old	Mean (SD) = 24.5 (6.7) years old, median = 26 years old, 95% CI: [23.7–25.2]	Time of treatment completion prior application submission: mean (SD) = 3.5 (4.6) years, median = 1.8 years, 95% CI: 3.0–4.0	Not reported	US census data from 2011 and 2013 using the groups “under age 35” and “25–34 years of age,” *n* = 16,513,000, and MEPS using the group “18–44 years of age,” *n* = 21,877,000	88%
Lim et al., 2020 [37]	Switzerland	Retrospective cohort study	*n* = 176	Not applicable	43.2% male	Not reported	Median (SD) age at treatment = 30.3 (±7.6) years old, range: 15.1–39.5 years old	Median time since treatment = 66 months, range: 12–236 months	All brain and skull base tumors	None	50%
Lu et al., 2021 [38]	USA	Cross-sectional study	*n* = 2588	NHIS (2010–2018) 64.2–82.0% for the different years	32.8% male	18–29 years old: 8.3%, 30–39 years old: 23.0%, 40–49 years old: 26.1%, 50–64 years old: 27.4%, 65–80 years old: 12.2%, 81+ years old: 2.9%	Median (IQR) = 31 (26–35) years old	(Categories are not mutually exclusive) < 2 years: 8.4%, ≥2 years: 91.6%, > 6 years: 75%, >16 years: 50%, >31 years: 25.0%	Lymphoma: 7.8%, melanoma: 12.3%, testicular cancer: 5.5%, thyroid cancer: 9.1%, ovarian cancer: 7.3%, uterine cancer: 10.8%, leukemia: 1.9%, breast cancer: 15.7%	Adults without cancer history (*n* = 256,964)	88%
Mader et al., 2017 [19]	Switzerland	Cross-sectional study	*n* = 160	41.10%	61.3% male	Mean (SD) = 33.5 (5.9) years old, 20–29 years old: 26.9%, 30–29 years old: 53.1%, ≥40 years old: 20%	Mean (SD) = 21.1 (2.9) years old, range: 16–25 years old, 16–20 years old: 43.8%, 21–25 years old: 56.3%	Mean (SD) = 11.9 (4.7) years	Lymphoma: 37.5%, germ cell tumor: 28.8%, CNS tumor: 9.4%, soft tissue sarcoma: 9.4%, leukemia: 8.1%, bone tumor: 3.8%, renal tumor: 1.9%, neuroblastoma: 1.3%	Swiss Health Survey (SHS), participants aged 20–50 years old, residents in the Canton of Zurich (*n* = 999)	100%
Meernik et al., 2020 [25]	USA	Cross-sectional study (restricted to working (full/part-time) at time of diagnosis)	*n* = 1328	12.80%	All female	Median (SD) = 41.0 (6.2) years old	Median (SD) = 34.0 (5.1) years old, range: 16–39 years old	Median (SD) = 7.0 (3.6) years, range: 3–15 years	Breast: 41.7%, thyroid: 22.3%, melanoma: 14.4%, lymphoma: 10.4%, gynaecologic (cervical, uterine, ovarian): 11.2%	None	100%
Nord et al., 2015 [39]	Sweden	Retrospective cohort study	*n* = 2146	Not reported	All male	Not reported,	Median = 32 years old, range: 18–60 years old	Follow-up for study: median = 10 years, range: 2–19 years	All testicular	General population without a cancer history (*n* = 8448)	100%
Nugent et al., 2018 [40]	USA	Cross-sectional study	*n* = 23	Not reported	69.9% male	Mean (SD) = 23.8 (4.0) years old, median (IQR) = 22.6 (5.0) years old	Mean = 17.4 years old, range: 15–21 years old, length of treatment: mean = 1.2 years	≥2 years since active cancer treatment	Hodgkin lymphoma: 43.4%, acute lymphoblastic leukemia:17.4%, Ewing’s sarcoma: 8.7%, osteosarcoma: 8.7%, germ cell tumor: 8.7%, acute myelocytic leukemia: 4.3%, chondrosarcoma: 4.3%, non-Hodgkin lymphoma: 4.3%	Controls were matched to the cancer survivors, being of the same gender and within 2 years of the survivor’s age (*n* = 14)	88%
Parsons et al., 2012 [17]	USA	Cohort study	*n* = 463 (all AYA cancer survivors)	Initial survey: 43.4%, follow-up survey: 88.7%	AYA cancer survivors working or in school full-time before diagnosis (*n* = 388): 64% male	Not reported	AYA cancer survivors working or in school full-time before diagnosis (*n* = 388): 15–19 years old: 13.1%, 20–24 years old: 17.8%, 25–29 years old: 24.7%, 30–34 years old: 23.2%, 35–39 years old: 21.1%	AYA cancer survivors working or in school full-time before diagnosis (*n* = 388): 15–19 months: 13.1%, 20–24 months: 42.5%, 25–29 months: 34%, 30–35 months: 10.1%, range: 25–35 months	Germ cell: 40.5%, Hodgkin’s lymphoma: 26%, non-Hodgkin’s lymphoma: 24.2%, sarcoma: 4.6%, acute lymphoblastic leukemia: 3.9%	AYA cancer survivors 15–24 months after diagnosis and working or in school full-time before diagnosis (*n* = 216)	100%
Strauser et al., 2010 [41]	USA	Longitudinal study (restricted to AYACS who were unemployed at time of application for vocational services)	*n* = 368	Not reported	57% male	Mean (SD) = 21.46 (2.39) years old, range: 18–25 years old	Not reported	>2 years	Not reported	None	63%
Sylvest et al., 2022 [42]	Denmark	Register-based cohort study	*n* = 4222	Not applicable	100% male	≥ 35 years	Range: 0–29 years,	CNS cancer: mean (SD) = 14.59 (9.30) years, hematological cancer: mean (SD) = 16.68 (10.67) years, solid cancer: mean (SD) = 9.37 (8.47) years	CNS tumors: 5.0%, hematological tumors: 6.5%, solid tumors: 88.5%	Age-matched comparison group of the general population (*n* = 794,589)	100%
Tangka et al., 2020 [43]	USA	Cross-sectional study	*n* = 830	28.40%	All female	Not reported	18–34 years old: 39.5%, 35–39 years old: 60.5%	Not reported	All breast cancer	None	100%
Tebbi et al., 1989 [44]	USA	Cross-sectional study	*n* = 40	30%	40% male	Mean (SD) = 26.4 (4.2) years old, range: 18–35 years old	Mean = 16.15 years old, range: 13–19 years old	Mean (SD) = 10.1 (3.2) years	Hodgkin’s/non-Hodgkin’s lymphoma: 47.5%, soft tissue sarcoma/melanomas: 20.0%, leukemia: 7.5%, bone tumors: 20.0%, ovarian/testicular: 5.0%	15 male and 25 female controls without a cancer history and with age range from 18 to 35 years old (*n* = 40)	88%
Thom et al., 2021 [45]	USA	Cross-sectional study	*n* = 212	65%	8.9% male	Mean (SD) = 35.3 (5.25) years old	Mean (SD) = 27.4 (7.17) years old	Mean (SD) time since treatment = 6.2 (5.89) years	Breast: 27.8%, lymphoma: 16.5%, colorectal: 11.3%, leukemia: 9.4%, brain: 7.1%, gynecological: 6.1%, sarcoma: 6.1%, thyroid: 4.7%, other: 8.0%, prefer not to respond: 0.5%	None	88%
Yanez et al., 2013 [46]	USA	Cross-sectional study	*n* = 106	66.50%	31.6% male	Mean (SD) = 32.2 (5.1) years old	Not reported	Range: 25–60 months, 3 years after treatment completion: 41%, 4 years after treatment completion: 31%, 5 years after treatment completion: 28%	Breast: 24.8%, cervical: 11.5%, melanoma: 9.7%, lung: 8.0%, colorectal: 3.5%, thyroid: 9.7%, testicular: 4.4%	AYA cancer survivors 0–24 months after diagnosis (*n* = 216)	88%

Abbreviations: d, diagnosis; s, study; t, treatment; fu, follow-up; CI, confidence interval; IQR, interquartile range; SD, standard deviation; NHIS, National Health Interview Surveys; MEPS, Medical Expenditure Panel Survey; CNS, central nervous system; RM, Malaysian ringgit.

**Table 2:** Characteristics of included qualitative studies.

First Author, Publication Year	Country	Study Design or Approach, Analysis Method	Sample Size	Gender: Percentage Male	Age at Time of Study	Age at Diagnosis	Time Since Diagnosis	Cancer Types	Study Quality
An et al., 2019 [47]	South Korea	Grounded theory/thematic analysis	*n* = 14	21.43% male	Range: 14–22 years old	Not reported	Not reported; adolescents who visited a hospital for follow-up care following treatment for leukemia	Acute lymphoid leukemia: 42.9%, acute myeloid leukaemia: 50%, chronic myeloid leukemia: 7.1%	80%
Brauer et al., 2017 [48]	USA	Grounded theory; systematic yet flexible coding process	*n* = 18	61.1% male	Mean = 26 years old, range: 19.8–34.6 years old	Age at treatment: mean = 23.3 years old, range: 18.5–29.7 years old	Time since treatment: mean = 32.8 months, range: 8–60 months	Acute myeloid leukemia: 56%, acute lymphoblastic leukemia: 28%, Hodgkin’s lymphoma: 11%, non-Hodgkin’s lymphoma: 5%	70%
Drake et al., 2019 [49]	Canada	Phenomenology; thematic analysis	*n* = 5	40% male	Mean (SD) = 32 (6.78) years old, range: 25–40 years old	Range: 18–39 years old	Not reported	5 participants with Hodgkin’s lymphoma, multiple myeloma, malignant neoplasm of the pineal region, thyroid cancer, and appendix cancer	80%
Elsbernd et al., 2018 [50]	Denmark	Thematic analysis	*n* = 9	22.2% male	Mean = 24.2 years old, median = 25 years old, range: 19–27 years old	Range: 17–24 years old	Time since last treatment: range: < 1–> 10 years	9 participants with lymphoma (2), breast (2), leukemia, cervical, testicular, pancreatic, and brain tumor	50%
Ghazal et al., 2021 [51]	USA	Cross-sectional study	*n* = 40	36.5% male	Not reported	Median (SD) = 28 (5.26) years old, range: 20–38 years old	Range: 1–5 years	Lymphoma: 82.5%, leukemia: 17.5%	90%
Gupta et al., 2020 [52]	USA	Thematic analysis combined with an abductive approach	*n* = 52	59.6% male	Mean (SD) = 25.29 (2.88) years old, range: 18–29 years old	Not reported	Mean (SD) = 31.25 (17.12) months	Hematologic: 61.5%, testicular: 38.5%	70%
Kent et al., 2012 [53]	USA	Hermeneutic phenomenology (interpretative method); grounded theory; narrative analysis	*n* = 19	52.6% male	15–19 years old: 5.3%, 20–23 years old: 10.5%, 24–26 years old: 15.8%, 27–29 years old: 15.8%, 30–33 years old: 26.3%, 34–36 years old: 21.1%, 37–39 years old: 5.3%	15–19 years old: 15.8%, 20–23 years old: 21.1%, 24–26 years old: 21.1%, 27–29 years old: 21.1%, 30–33 years old: 10.5%, 34–36 years old: 10.5%	Range: 6 months–6 years	Non-Hodgkin’s lymphoma: 21.1%, Hodgkin’s: 10.5%, brain tumor: 10.5%, acute lymphoblastic leukemia: 10.5%, ovarian: 10.5%, melanoma: 5.3%, Wilm’s tumor: 5.3%, testicular: 5.3%, ovarian: 5.3%, acute lymphoblastic leukemia: 5.3%, multiple myeloma: 5.3%, aplastic anemia: 5.3%	60%
Magrath et al., 2021 [54]	United Kingdom	Phenomenological analysis, analysis was performed iteratively	*n* = 8	50% male	Mean = 21.8 years old, median = 21 years old, range: 18–27 years old	Mean = 17.6 years old, median = 17.5 years old, range: 16–19 years old	Not reported	Brain tumor: 12.5%, lymphoma: 75%, leukemia: 12.5%	90%
Parsons et al., 2008 [55]	Canada	Postmodern narrative approach; data analysis occurred in conjunction with data collection	*n* = 14	57.1% male	Mean = 27.4 years old, median = 26.5 years old, range: 18–38 years old	Mean = 24.2 years old, median = 23 years old, range: 16–35 years old	Range: 1–6 years	All osteosarcoma	70%
Raque-Bogdan et al., 2015 [56]	USA	Consensual method	*n* = 13	All female	Range: 24–43 years old	Mean (SD) = 30 (5) years old, median = 27 years old, range: 21–38 years old	Mean = 3.54 years	All breast	80%
Stone et al., 2019 [57]	USA	Constructivist grounded theory; analytic techniques including initial, focused, axial, and theoretical coding procedures	*n* = 12	25% male	Mean = 43.9 years old, range: 28–59 years old	Mean = 29 years old, 18–29 years old: 50% 30–39 years old: 50%	Mean = 14.8 years, range: 8–35 years	Breast: 33%, leukemia or lymphoma: 33%, melanoma: 8%, testicular: 317%, thyroid: 8%	90%

Abbreviations: d, diagnosis; s, study; t, treatment.

## 3. Results

### Literature Search and Study Characteristics

**Figure 1:**
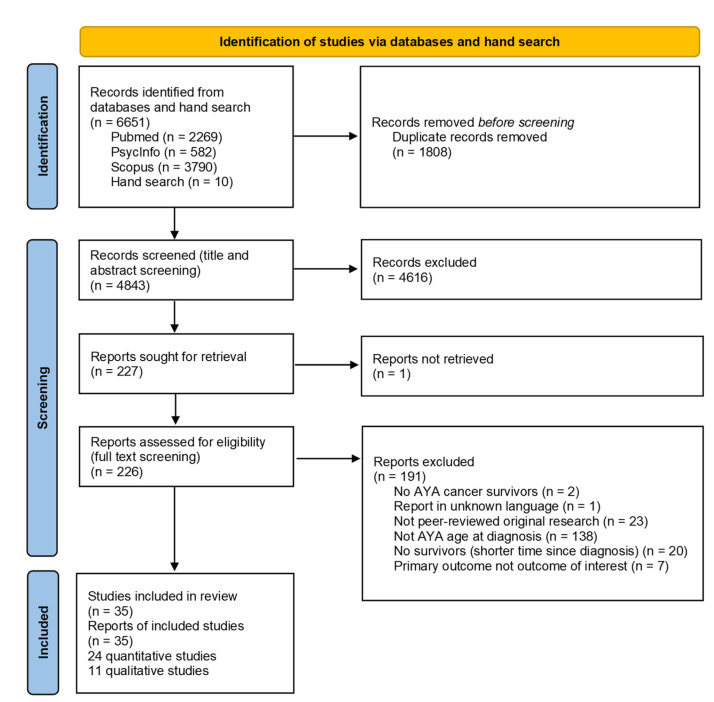
PRISMA flow diagram of included studies.

While searching the three databases, 6651 articles were identified, and finally, 35 articles were included [17,19,20,23,24,25,29,30,31,32,33,34,35,36,37,38,39,40,41,42,43,44,45,46,47,48,49,50,51,52,53,54,55,56,57] (Figure 1). We included 24 quantitative (Table 1) and 11 qualitative (Table 2) studies. The majority of the studies were conducted in North America (24, 69%), nine in Europe (26%), and two in Asia (6%). Fourteen of the quantitative studies (58%) studies included a comparison group. The majority of the studies (29, 83%) included different types of cancer. Variations in sample size (quantitative studies: 23–4′222, qualitative studies: 5–52), age at diagnosis or study, and time since diagnosis were observed. Three articles reported only on education outcomes, nine only on employment outcomes, and eight only on financial outcomes. Another six articles described both education and employment outcomes, and nine studies addressed both employment and financial outcomes.

## 4. Impact of Cancer

### 4.1. Education

After being diagnosed with cancer, many AYA cancer survivors experienced a disruption in education [46,48,50] (Table 3). In one study, AYA cancer survivors reported having kept up with school via the Internet while being treated for cancer [47]. Those who left school for cancer treatment wanted to return to school as quickly as possible to keep up with peers but also for a sense of normalcy [47,48,50]. In doing so, they experienced enormous hurdles and challenges, some related to experiencing late effects such as fatigue [54]. Problems arose, especially in re-entry, which could only occur at the beginning of a school year [48,50]. AYA cancer survivors reported different educational pathways compared to the general population: More had completed upper secondary school and fewer university education in Switzerland [19]. In Germany, survivors were more likely to have attended high school, whereas rates of college and university graduation were similar [20]. Survivors of CNS cancer were less progressed in their education compared to age-matched comparisons [42]. On the other hand, survivors of hematological and solid cancers reached higher educational levels [42].

A stay in the intensive care unit (ICU) during treatment, experiencing visual or hearing late effects, and having a migration background were identified as characteristics associated with lower education [19,20].

### 4.2. Employment

In most studies investigating employment, the majority of AYA cancer survivors were employed at the time of the study [17,19,20,24,25,31,32,34,35,37,40,41,42,43,44,46] (Table 4). Some survivors reported reduced ability to work and were consequently uncertain whether cancer had long-term effects on their ability [31,49]. Compared to before their cancer diagnosis, more survivors were unemployed after their cancer treatment (19% before treatment, 38% six months after treatment [30]; from 9.5% to 23.8% pre- and post-treatment, respectively [37]), about half of survivors reported paid or unpaid time off, and about 10% of survivors quit or lost their job at diagnosis [43]. In most studies comparing survivors with other populations, there was no difference between the employment rates in survivors and the comparison group [19,20,31,40,44]. One study reported that slightly more AYA cancer survivors were outside the workforce compared to the comparison group [42]. Survivors started being engaged in paid employment at an older age compared to the general population [20]. In one study, AYA cancer survivors were significantly less likely to be employed than the comparison group [34]. In two studies from the USA, AYA cancer survivors reported experiencing employment disruption [25,46]. Breast cancer survivors reported stopping working was impossible due to financial hardship or insurance needs [56]. About half of the survivors preserved employment in the same workplace as before the diagnosis [55,57]. For others, the cancer diagnosis meant a change of perspective, be it that they changed their workplace [50,55,56] or that they reported that the meaning of work had changed [51]. Their cancer diagnosis was seen as a catalyst for a change of career and thus an inspiration for a new beginning [49].

Longer time since first cancer diagnosis [31], younger age at diagnosis [19,20], female gender [19,31], lower education [19,31], and experiencing late effects or impaired health [19,20,31] were identified as characteristics associated with unemployment. In another study, with a longer time since end of treatment, the percentage of AYA cancer survivors being unemployed decreased [30].

### 4.3. Financial Outcomes

Two studies addressed the income of AYA cancer survivors and compared it to the general population [34,36,44] (Table 5). In an early study, AYA cancer survivors had a higher income than the general population [44]. This difference may reflect a strong motivation to achieve higher goals among survivors [44]. In a more recent study, more AYA cancer survivors had a low family income and fewer survivors had a high family income [34]. AYA cancer survivors reported a negative net worth, whereas young adults from the general population reported a positive net worth [36]. Indirect medical costs were reported in three studies, with AYA cancer survivors having reported more missed work days than the comparison group in all studies [32,34,39]. AYA cancer survivors were significantly more likely to experience medical financial hardship compared to adults without a cancer history [29,38], and survivors reported a high level of financial toxicity (financial-related hardship) [45]. About half of the women with breast cancer experienced a financial decline due to their cancer diagnosis [43]. Three Scandinavian studies reported on disability pension uptake [31,33,39]. Compared with the general population, AYA cancer survivors received disability pensions at similar rates [31]. Compared with childhood cancer survivors, AYA cancer survivors were less likely to receive disability pensions [33].

Older age at time of study [36], chemotherapy and radiation [24,39], lower education [43,45], psychological distress [29], and more chronic conditions [23] were identified as characteristics associated with a higher financial burden. AYA cancer survivors with more chemotherapy courses were more likely to receive a disability pension [39].

### 4.4. Study Quality

Although some studies were designed as longitudinal or cohort studies, outcomes were cross-sectionally assessed. The average quality rating for cross-sectional studies (mean = 90%, range: 50–100%; Table 1) was slightly higher than for qualitative studies (mean = 75%; range: 50–90%; Table 2). No conclusive patterns in reported outcomes by study quality were identified.

## 5. Discussion

With this systematic review, we showed that a cancer diagnosis in adolescence or young adulthood significantly impacted educational, employment, and financial outcomes. Re-entry to school or work after cancer treatment was challenging. After treatment, most survivors were employed but started their employment at an older age than the general population. Overall, no disadvantages in income were found. Survivors reported more absent work days than the comparisons. The main determinants for adverse outcomes were female gender, younger age at diagnosis, chemotherapy and radiotherapy, and experiencing late effects.

Our systematic review is in line with the findings of a previous review on work-related issues in AYA cancer survivors [58]. For many AYA cancer survivors, the cancer diagnosis interrupted their current engagement at school or work. This interruption delayed the attainment of education and work goals and sometimes forced survivors to rely on social security benefits or file for bankruptcy. This did not mean that AYA cancer survivors could not achieve a successful career compared to healthy controls, but they did start the career later. Many survivors were willing to return to school or work, although cancer treatment and its side effects often imposed hurdles. Our review showed that these long-term consequences forced some AYA cancer survivors to wait a certain amount of time to return to school, or for formerly employed survivors, it meant a change of workplace. Whereas some AYA cancer survivors perceived working as a return to normalcy, others described a change in perspective and redefined their professional careers.

One study found that AYA cancer survivors earned more compared to the general population [44]. One reason could be the change in perspective leading to a job change, possibly resulting in survivors earning more than they did before diagnosis [59,60]. For instance, jobs with less physical effort might be, on average, better paid compared to jobs with more physical effort involved. Within AYA cancer survivors, financial outcomes varied with age at the time of the study. Although older survivors earned more [36], as seen in the general population, the study also found that older survivors reported a more severe financial impact [36]. Whereas older survivors were more likely to be married and thus had a potential additional source of income through their partner, they received less parental support, were more likely to have dependent children, and were more likely to own a home compared to younger survivors, indicating the need for more financial resources for older survivors. AYA cancer survivors diagnosed with breast cancer missed more work days and home productivity days (spending more than half of the day in bed due to illness) compared to women without breast cancer, resulting in higher indirect productivity costs [32].

According to this review, AYA cancer survivors diagnosed at a younger age were found to be particularly vulnerable to adverse outcomes. One explanation for the lower educational attainment might be that they were still pursuing education and could not keep up with fellow students due to the interruption caused by cancer [47]. Unemployment might be higher because they may prioritize their health over their career [56,61]. Health insurance is organized differently in different countries. In countries where health insurance is not mandatory or related to employment, an explanation for the high financial burden might be that AYA cancer survivors were believed to be too young to need health insurance before the cancer diagnosis.

Chemotherapy and radiotherapy and a stay in the ICU during treatment were found to be determinants for adverse outcomes in all domains studied [20,39]. ICU stays are costly and associated with an increased number of potentially life-threatening complications that can negatively impact patient prognosis [62,63]. This could prolong their absence from school and work and affect their financial situation in the long run.

**Figure 2:**
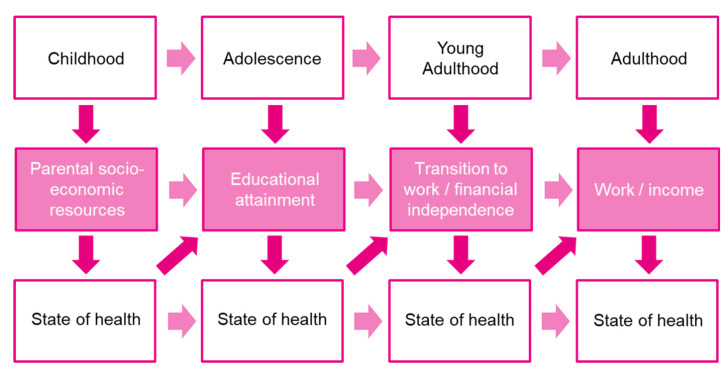
Dynamic interaction between life stages and state of health (own adaptation, based on (Adler et al., 2007 [64]; Fardell et al., 2018 [18]).

Our three outcomes of interest, i.e., education, employment, and financial outcomes, are linked to the different life stages (Figure 2). Whereas educational attainment is the primary focus in adolescence, transitioning to work and gaining financial independence becomes more important in young adulthood. However, all stages of life have one aspect in common: a reciprocal relationship with the state of health. If the state of health is deteriorating, this affects the current stage of life and is also likely to have long-term consequences for the following stage of life. Therefore, it is important to consider these three outcomes as mutually dependent rather than independent factors, also in the case of a cancer diagnosis in adolescence or young adulthood. Taking a holistic approach and considering the reciprocal relationship between outcomes and state of health can ensure a successful career even after a cancer diagnosis in adolescence or young adulthood.

## 6. Limitations and Strengths

Countries have different education, labor, and financial systems. Furthermore, there were significant differences in how the data were collected. This made comparisons across studies challenging. Most of the included studies were based on self-reported data. For these studies, self-report bias might be present. As for other systematic reviews, there is a potential for language and publication bias. We included publications in English and other languages known to the research team (only one publication written in Japanese had to be excluded) and published in the three databases searched.

The comprehensive literature review (search in three relevant databases) is a strength of the study. For this systematic review, over 5000 articles were screened for eligibility. Each article was screened independently by two reviewers, and three reviewers were involved in the decision process. The comprehensive search allowed for the inclusion of studies from different countries with different educational, employment, and financial contexts. The three outcomes were purposely chosen to represent a life course perspective. The carefully selected, mutually exclusive, and collectively exhaustive search terms ensured that we were able to include relevant studies, including a broad range of AYA cancer survivors, different cultural backgrounds, the whole AYA age range at diagnosis, and different time phases after diagnosis. Extensive hand searching and the search update ensured that the most recent articles and articles that would have been missed with the search in the databases were included as well.

## 7. Implications

Identifying AYA cancer survivors at risk for adverse educational, employment, and financial outcomes is important for developing tailored support strategies for cancer patients and survivors throughout their whole cancer trajectory. We found that most survivors returned to school or work after cancer treatment. However, this re-entry was associated with difficulties and hurdles. To enable a successful return to school or work, AYA cancer survivors should be supported in navigating the system [65] and involve key persons such as peers, teachers, or employers, and employees should be informed and supported as well [66]. Flexible working conditions might help survivors with successfully returning to work [67] and being able to stay in the workforce in the long term. Survivors in their last years of school or their first years of employment might be especially vulnerable to adverse effects on their education and employment. Individual support options focusing on cancer- and treatment-related impairments as well as abilities and potential new directions for their employment should be provided [67]. Furthermore, open conversations about finances should be held with AYA cancer patients and survivors. Such conversations can empower patients and survivors and increase their knowledge about existing financial assistance services. Further research should be done in the area of insurance at a young age. Where health insurance is optional, young people often think they are too young for insurance [68], as chronic illness may affect them less frequently than older people.

Although most AYA cancer survivors experience some degree of negative impact of their diagnosis on education, employment, or financial outcomes, many survivors also do well. It might be worth looking at their strategies to overcome the challenges of a cancer diagnosis during adolescence or young adulthood and to re-enter school or work successfully.

Most of the included studies were of a cross-sectional design. In future research, longitudinal studies in AYA cancer survivors could expand the understanding of the impact of cancer diagnosis and treatment throughout the cancer trajectory. Multiple measurement time points could be used to assess the individual courses of AYA cancer survivors. These results might expand the knowledge on appropriate time points for tailored support to AYA cancer survivors to mitigate their risk for adverse education, employment, and financial outcomes and improve their well-being.

## 8. Conclusions

Although most AYA cancer survivors were able to re-enter education and employment, they reported difficulties with re-entry and delays in their employment pathway. We found some determinants for adverse outcomes, but the results were heterogeneous. To facilitate successful re-entry, age- and situation-tailored support services along the cancer trajectory should be developed and implemented to prevent future social inequalities and adverse educational, employment, and financial outcomes in the long term.

## Figures and Tables

**Table 3:** Impact of cancer on education outcomes in adolescent and young adult (AYA) cancer survivors.

First Author, Publication Year	Measurements for Education Outcomes	Education Outcomes	Determinants for Adverse Education Outcomes (Quantitative Studies) or Selected Citations (Qualitative Studies, Indicated in Italics)
An et al., 2019 [47]	Difficulties in school, difficulties in returning to school	Identified themes: feelings of alienation from friends, difficulty in studying, stuck being different from others, apologetic feelings for family, feelings of having an uncertain future	*“I had a university and major in mind, but after an absence from studying for two years, it was very hard to catch up within one year. I put in a great deal of effort in that respect, but it was very difficult.” (Female, 22 years old)*
Brauer et al., 2017 [48]	Resuming work and school after hematopoietic cell transplantation	Identified themes: rushing to resume school/work, motivating factors, barriers to successful and sustainable re-entry	*“I had to withdraw from that whole semester, that whole year that I was there. And pay the fee of attending the school when I didn’t even get credit for being there, because I missed finals. [...] It was basically, ‘Hey, you missed finals. That’s how our grading system works. There’s no exception about it. And here’s your five, ten thousand dollar fee that you owe’.”*
Dieluweit et al., 2011 [20]	High school attainment, professional training, college or university degree	AYA cancer survivors vs. comparison group: high school attainment: 52.4% vs. 28.3% (Cramer’s V = 0.139, *p* < 0.001), professional training: 85.2% vs. 85.9% (Cramer’s V = 0.009, not significant), college/university degree: 24.7% vs. 17% (Cramer’s V = 0.093, *p* = 0.001)	High school degree: stay in an intensive care unit (OR = 0.73, CI = 0.54–0.99, *p* = 0.042), visual or hearing late effects (OR = 0.69, CI = 0.48–0.99, *p* = 0.048) college/university degree: higher age at time of study (OR = 1.08, CI = 1.05–1.11, *p* < 0.001), female gender (OR = 0.67, CI = 0.48–0.95, *p* = 0.0025), CNS tumor (reference: leukemia and lymphoma) (OR = 0.39, CI = 0.17–0.92, *p* = 0.0031), neuropsychological late effects (OR = 0.5, CI = 0.27–0.91, *p* = 0.024)
Elsbernd et al., 2018 [50]	Management of returning to secondary or higher education	Identified themes: symptoms and late effects, navigating the system, lack of understanding from peers, unofficial support, changed perspectives	*“I think you get a little guidance, but then you are on your own.” (Female, 24 years old)*
Mader et al., 2017 [19]	Educational achievement	AYA cancer survivors vs. comparison group: basic education: 8.2% vs. 4.8%, vocational training/apprenticeship: 46.5% vs. 47.2%, upper secondary education: 33.3% vs. 26.7%, university education: 11.9% vs. 21.3%, (*p* = 0.012 for educational achievement)	Only basic education: migration background (OR = 10.23, CI = 4.64 to 22.55, *p* < 0.001)
Magrath et al., 2021 [54]	Experiences while returning to education	Identified themes: late effects, systems, adjusting to losses, mechanisms facilitating resilience	*“The difficulty concentrating was the single most difficult aspect of the cancer because I couldn’t look at a screen, I couldn’t look at my phone, I couldn’t look at a laptop, I couldn’t do some work, I couldn’t even do a powerpoint.”* *“They put me in for the exam on a different day, they also gave me longer time, in exams, which was useful”. (AYA4)* *“I guess I was concerned about just not being able to go to uni, umm, it’s always been a plan to go and study [.] so I was concerned about the realisation that maybe that wouldn’t be a possibility.”* *“I had help from the charity CLIC, they helped arrange for me to go back to university so they arranged with my lecturers to skype me into the lectures as opposed to me physically going in.”*
Parsons et al., 2012 [17]	Full-time work or school participation, belief of cancer leading to a negative impact	Results for the 388 AYA cancer survivors who had been working or in school full-time before diagnosis: full-time work or school participation: 15–19 months since diagnosis: 74.0% full-time or work at follow-up, 20–24 months since diagnosis: 75.8% full-time or work at follow-up, 25–29 months since diagnosis: 69.9% full-time or work at follow-up, 30–35 months since diagnosis: 66.7% full-time or work at follow-up Belief: 15–19 months since diagnosis: 44.0% negative impact on plans, 20–24 months since diagnosis: 33.9% negative impact on plans, 25–29 months since diagnosis: 30.8% negative impact on plans, 30–35 months since diagnosis: 38.5% negative impact on plans	-
Sylvest et al., 2022 [42]	Progression in the educational system	Survivors vs. comparison group: Survivors of CNS cancer had lower odds of having progressed in the educational system than those from the age-matched comparison group: high school: aOR = 0.25; 95% CI: 0.11–0.58; vocational training: aOR = 0.58, 95% CI: 0.42–0.80; short-term further education: aOR = 1.17, 95% CI: 0.71–1.93; medium-term further education: aOR = 0.35, 95% CI: 0.19–0.65; long-term further education: aOR = 0.88, 95% CI: 0.57–1.36. Survivors of hematological and solid cancers showed an opposite trend, with higher odds of progressing to higher educational levels compared to the comparison group: high school: aOR = 0.76; 95% CI: 0.41–1.41 and aOR = 1.00, 95% CI: 0.86–1.16; vocational training: aOR = 0.96, 95% CI: 0.70–1.32 and aOR = 1.07, 95% CI: 0.98–1.16; Short-term further education: aOR = 0.98, 95% CI: 0.59–1.61 and aOR = 1.12, 95% CI: 0.98–1.28; medium-term further education: aOR = 1.15, 95% CI: 0.82–1.62 and aOR = 1.17, 95% CI: 1.07–1.29; long-term further education: aOR = 1.17, 95% CI: 0.84–1.63 and aOR = 1.17, 95% CI: 1.07–1.28.	Cancer type: The percentage of men who attained primary school only was higher in survivors of CNS cancer (36%) than in men with hematological cancer, solid cancer, or no cancer diagnosis (19%, 18%, and 20%, respectively). The opposite was true for medium-term and long-term further education. Age at diagnosis: The percentage of primary school as the highest educational attainment was slightly higher in men diagnosed with cancer when they were 0–9 years old (23%) than in men who were older at diagnosis (10–19 years: 20%, 20–29 years: 19%). Diagnosis decade: This percentage for primary school was also higher in men diagnosed with cancer between 1978 and 1989 (24%) than in those diagnosed in later decades (1990–1999: 18%, 2000–2009: 14%). Contrasting associations were observed for long-term further education (1978–1989: 12%, 1990–1999: 13%, 2000–2009: 20%).
Yanez et al., 2013 [46]	Educational attainment, cancer-related education/work interruption	Educational attainment: 41.6% of AYA cancer survivors reported an educational attainment of less than a college degree. Cancer-related education/work interruption: 62.3% of AYA cancer survivors reported an interruption in education or work.	Time since diagnosis: AYA cancer survivors 25–60 months since diagnosis vs. 13–24 months since diagnosis vs. 0–12 months since diagnosis: Educational attainment: 41.6% vs. 34.3% vs. 39.2% Cancer-related education/work interruption: 62.3% vs. 56.1% vs. 66.1%

Abbreviations: OR, odds ratio; CI, confidence interval; *p*, *p*-value; UK, United Kingdom; aOR, adjusted odds ratio; CNS, central nervous system; AYA, adolescent and young adult.

**Table 4:** Impact of cancer on employment outcomes in adolescent and young adult (AYA) cancer survivors.

First Author, Publication Year	Measurements for Employment Outcomes	Employment Outcomes	Determinants for Adverse Employment Outcomes (Quantitative Studies) or Selected Citations (Qualitative Studies, Indicated in Italics)
Bhatt et al., 2021 [30]	Employment status	Employment status: The percentage of full-time employed survivors was lower 6 months after HCT treatment than before treatment, whereas the rates for part-time employment, unemployment, or medical disability were higher 6 months after treatment than before treatment. Before treatment: full-time 43%, part-time 4%, unemployed 19%, medical disability 16%, unknown 17% 6 months after treatment: full-time 18.3%, part-time 6.9%, unemployed 38.2%, medical disability 36.6%, unknown 0%	Time after treatment: The percentages of survivors working full- or part-time increased with time after treatment (full-time: from 18.3% at 6 months to 50.7% at 3 years; part-time: from 6.9% at 6 months to 10.5% at 3 years). The percentages for unemployment and medical disability decreased over time after treatment (unemployment: from 38.2% at 6 months to 18.3% at 3 years; medical disability: from 36.6% at 6 months to 21% at 3 years).
Brauer et al., 2017 [48]	Resuming work and school after hematopoietic cell transplantation	Identified themes: rushing to resume school or work, motivating factors, barriers to successful and sustainable re-entry	-
Dahl et al., 2019 [31]	Employment status, work ability (current work ability compared to the lifetime best)	Employment status: 75.5% of AYA cancer survivors were employed. Work ability: 62% of AYA cancer survivors reported high current work ability. Mean work ability among employed (8.3) vs. unemployed (3.9) AYA cancer survivors AYA cancer survivors vs. comparison group: Employment status: survivors (m = 83%, f = 73%) vs. Norwegian population (m = 81%, f = 76%) Disability pension recipient: AYA cancer survivors (m = 10%, f = 19%) vs. Norwegian population (m = 11%, f = 13%)	Unemployment: longer time since first cancer diagnosis (OR = 1.03, CI = 1.01–1.05, *p* = 0.002), increased mean number of adverse events (OR = 1.21, CI = 1.16–1.26, *p* < 0.001), female gender (OR = 1.77, CI = 1.28–2.46, *p* = 0.001), low basic education (OR = 2.52, CI = 1.92–3.3, *p* < 0.001), comorbid cardiovascular disease (OR = 1.85, CI = 1.31–2.63, *p* = 0.001), decreased general health (OR = 0.98, CI = 0.97–0.98, *p* < 0.001), increased level of depression (OR = 1.18, CI = 1.15–1.22, *p* < 0.001)
Dieluweit et al., 2011 [20]	Employment status	AYA cancer survivors vs. comparison group: employment rate: 79.6% vs. 74.2% (Cramer’s V = 0.064, *p* = 0.013)	Employment: higher age at time of study (OR = 1.04, CI = 1.01–1.08, *p* = 0.017), female (OR = 0.59, CI = 0.34–0.89, *p* = 0.016), having children (OR = 0.36, CI = 0.23–0.56, *p* < 0.001), having neuropsychological late effects (OR = 0.55, CI = 0.34–0.89, *p* = 0.0016)
Drake et al., 2019 [49]	Perspectives on and experiences with return to work following treatment	Identified themes: uncertainty about return to work, cancer as a catalyst for a career change, importance of employment benefits, benefit of YA-specific resources	“Ahh because my current role in the [company] is meaningless and repetitive I’d be happy to leave that company... people they, they want to do something that’s meaningful. To come through this experience and it kind of ahh turns their world upside down, wakes them up in some ways. They have an awakening and ahh *pause* in my case I guess I have to do something. I have to do work that is meaningful, which is why I’m exploring this opportunity with [company].”
Ekwueme et al., 2016 [32]	Employment status, work days lost, home productivity days lost	Employment status: 75.43% of AYA cancer survivors employed Work days and home productivity days lost: AYA cancer survivors missed 19 work days and 17 home productivity days. AYA cancer survivors vs. women aged 18–44 without breast cancer: Employment status: employed: 75.43% vs. 78.38% Workdays and home productivity days lost: AYA cancer survivors missed more work days (19 days vs. 4 days, *p* < 0.01) and home productivity days (17 days vs. 4 days, *p* < 0.01).	-
Ghazal et al., 2021 [51]	Perspectives of work-related goals	Identified themes: self-identity and work, perceived health and work ability, financial toxicity	*“(…) in order to take care of myself, I had to quit this job that had been my end goal… I had to go back to the job that I had worked all through school... [with diagnosis and treatment] it’s taxing for me to do the job that I chose as my career, and then now I can’t even afford to do that job… despite everything I’ve done in my education to get to this point… I’m literally thinking to myself, “What have I been working my whole life for?”*
Guy et al., 2014 [34]	Functional limitations, employment status	Functional limitations: 17% of AYA cancer survivors experienced limitations at work, with housework, or in school; 11.9% were completely unable to work at a job, do housework, or go to school. Employment status: 33.4% of AYA cancer survivors were not employed; reasons for not being employed were retirement (41%), inability to work because of illness or disability (34.1%), and not being able to find work (20.7%) AYA cancer survivors vs. comparison group: Functional limitations: limitations in work, housework, or school: 17 vs. 10.5%, *p* < 0.001; being completely unable to work at a job, do housework, or go to school: 11.9 vs. 6.7%, *p* < 0.001 Employment status: not employed: 33.4% vs. 27.4%, *p* < 0.001	-
Hamzah et al., 2021 [35]	Employment status, career engagement and quality of working life	Employment status: 67.5% of AYA cancer survivors had permanent employment, 12.5% had temporary employment, 14.8% were self-employed, 5.2% worked part-time. Career engagement and quality of working life: positive correlation of career engagement with meaning of work (r = 0.578, *p* < 0.001), perception of the work situation (r = 0.665, *p* < 0.001), atmosphere in the work environment (r = 0.648, *p* < 0.000), understanding and recognition in the organization (r = 0.553, *p* < 0.001), negative correlation of career engagement with problems because of health situation (r = −0.688, *p* < 0.001), effect of disease and treatment (r = −0.656, *p* < 0.000)	-
Ketterl et al., 2019 [24]	Employment status, physical and mental impairment of work-related tasks, extended paid or unpaid time off from work	Employment status: 84.4% of AYA cancer survivors were employed. Physical and mental impairment of work-related tasks: Among employed survivors, 70.2% reported a physical component in their job and 58.6% reported that cancer interfered with physical tasks required by their job. A total of 54.2% reported that cancer interfered with their ability to perform mental tasks required by their job.	Treatment: Chemotherapy: inference with job-related physical tasks (OR = 1.97, CI = 1.22 to 3.11, *p* < 0.01), inference with mental tasks required by a job (OR = 3.22, CI, 2.15 to 4.79, *p* < 0.01), time off from work (OR = 3.56, CI = 2.31 to 5.47, *p* < 0.01), borrowing ≥ USD 10,000 (OR = 3.05, CI = 1.53 to 6.09, *p* < 0.01) compared with survivors who were not exposed to chemotherapy. Radiation: interference with job-related physical tasks (OR = 1.66, CI = 1.08 to 2.41, *p* < 0.05) compared with survivors who did not receive radiation. Surgery: extended paid time off from work (OR = 0.54, CI = 0.54 to 1.00, *p* < 0.05) compared with survivors who did not receive surgery.
Lim et al., 2020 [37]	Employment status	Employment status: pre- and post-treatment: unemployment: from 9.5% to 23.8%, employment with sick leave: from 14.3% to 0%, employment: from 42.9% to 63.5%, in education: from 33.3% to 12.7%	-
Mader et al., 2017 [19]	Employment status	AYA cancer survivors vs. comparison group: employment status: 91.2% vs. 89.5% (*p* = 0.515)	Unemployment: female gender (OR = 2.52, CI 1.36 to 4.68, *p* = 0.004), having only basic education (OR = 2.78, CI = 1.01 to 7.65, *p* = 0.048), being married (OR = 0.53, CI = 0.29 to 0.98, *p* = 0.042), younger age at diagnosis (OR = 5.29, CI = 1.32 to 30.79, *p* = 0.010), self-reported late effects (OR 4.70, CI = 1.26 to 19.49, *p* = 0.009)
Meernik et al., 2020 [25]	Employment status, employment disruption	Employment status: 17% part-time employment, 82.6% full-time employment Employment disruption: 32% of AYA cancer survivors reported an employment disruption, categorized as stopping work completely (14%), reducing work hours (12%), taking temporary leave (6%), or both a reduction in hours and temporary leave (5%).	-
Nord et al., 2015 [39]	Mean days of sick leave or disability pension	AYA cancer survivors vs. comparison group: Mean days of sick leave or disability pension: AYA cancer survivors having received no or limited treatment vs. comparisons: 3rd year after diagnosis: 16 vs. 14 days, 5th year after the diagnosis: 15 vs. 12 days AYA cancer survivors having received extensive treatment vs. comparisons: 3rd year after diagnosis: 26 vs. 14 days, 5th year after diagnosis: 23 vs. 12 days	Treatment intensity: Mean days of sick leave or disability pension: AYA cancer survivors having received no or limited treatment: 3rd year after diagnosis: 16 days, 5th year after diagnosis: 15 days AYA cancer survivors having received extensive treatment: 3rd year after diagnosis: 26 days, 5th year after diagnosis: 23 days
Nugent et al., 2018 [40]	Employment status, occupational function	AYA cancer survivors vs. comparisons: Employment status: full-time student, not working (17.4% vs. 21.4%); student and part-time work (21.7% vs. 28.6%); student and full-time work (4.3% vs. 0%); part time work only (13% vs. 0%); full-time work only (43.4% vs. 50%) Occupational function: no significant difference between AYA cancer survivors (mean score = 4.5 ± 5.28 [2.13–6.87]) and comparisons (mean score 4.67 ± 4.34), Cohen’s d = −0.034 [−0.78 to 0.72]	-
Parsons et al., 2008 [55]	Lived experiences of resuming vocational work	50% of AYA cancer survivors returned to their pre-illness occupation, whereas the other half were forced to change careers. Regardless of whether their professional status changed, all respondents recounted how their relationship with their vocation had been profoundly altered by the illness. Return to work was interconnected with aspects of life such as support (including financial), possession of disability and unemployment benefits, and entitlements to sick leave from employment/training/educational programs. All AYA cancer survivors expressed a strong desire to resume vocational pursuits but experienced returning to work as hard work. They portrayed themselves as “hard workers” due to drawing heavily on discourses of “work ethics.” Concerns were raised regarding financial pressures, but willingness to physically return was also expressed.	*“I’m afraid to apply for jobs, to be rejected. ‘Cause I could send my resume in, and I’m sure I’ll get an interview, but I go in there with my crutches or a cane, it’s like, even my brother-in-law was saying, “How much work can this person do for me?” (31 years old at diagnosis, 35 years old at interview)*
Parsons et al., 2012 [17]	Full-time work or school participation, belief of cancer leading to a negative impact	Results for the 388 AYA cancer survivors who had been working or in school full-time before diagnosis: Full-time work or school participation: 15–19 months since diagnosis: 74.0% full-time or work at follow-up, 20–24 months since diagnosis: 75.8% full-time or work at follow-up, 25–29 months since diagnosis: 69.9% full-time or work at follow-up, 30–35 months since diagnosis: 66.7% full-time or work at follow-up Belief: 15–19 months since diagnosis: 44.0% negative impact on plans, 20–24 months since diagnosis: 33.9% negative impact on plans, 25–29 months since diagnosis: 30.8% negative impact on plans, 30–35 months since diagnosis: 38.5% negative impact on plans	-
Raque-Bogdan et al., 2015 [56]	Effect of breast cancer on work lives and career development	Identified themes: cancer-related work challenges, coping with cancer-related work challenges, reappraisal of career development after cancer and components of career, components of career and life satisfaction after cancer	*“So the 2 months that I missed, it has slowed down my learning in my career at a time that learning is very important. Part of that is time away from work. But much of that is that I have not had the capacity to work as intensely at the level that is necessary.”*
Stone et al., 2019 [57]	Work experiences	Identified themes: process of revealing the survivor-self, process of sustaining work ability, process of accessing support	*“I was back working, you know, full-time, maybe 3 or 4, 5 days later.”*
Strauser et al., 2010 [41]	Competitive employment, use of vocational services	Competitive employment: 51.6% of AYA cancer survivors were competitively employed.	AYA cancer survivors using more services and spending more time in services were more likely to be employed. Employment was associated with the use of following services: vocational training (OR = 2.03, CI: 1.03 to 4.00), miscellaneous training (OR = 3.4, CI: 1.47 to 7.96), job search assistance (OR = 4.01, CI: 1.80 to 8.97), job placement assistance (OR = 2.24, CI: 1.11 to 4.52), on-the-job support (OR = 4.2, CI: 1.66 to 10.63), maintenance (OR = 2.85, CI: 1.38 to 5.90)
Sylvest et al., 2022 [42]	Being outside the workforce	AYA cancer survivors vs. comparison group: The percentage of cancer survivors being outside the workforce (retired/receiving transfer income) was higher (9%) than the percentage in the comparison group with no cancer diagnosis (6%).	-
Tangka et al., 2020 [43]	Employment status, work benefits at diagnosis, impact on employment status	Employment status: 73.4% of participants were employed at the time of diagnosis. Out of these, 64.9% worked for a private or non-profit organization; 21.0% for a branch of federal, state, or local government; and 7.5% were self-employed. Work benefits at diagnosis: The respondents reported that the following work benefits at diagnosis were available for them: paid sick leave: 55.1%, flexible scheduling: 49.4%, disability: 40.5%, unpaid sick leave: 36.8%, flexible location: 21.5%, none of the above: 10.9%. For most of the women, their employer was very supportive during treatment (66.8%). For the others, their employer was neutral or somewhat supportive (17.9%), unsupportive (5.5%), or unaware of the diagnosis (3.7%). Impact on employment status: Survivors reported that their diagnosis and treatment impacted their employment as follows: changed jobs within company: 5.4%, avoided changing jobs to keep health insurance: 23.5%, changed jobs to get health insurance: 1.5%, took paid time off: 55.1%, took unpaid time off: 47.3%, quit job: 12.2%, retired early: 1.2%, lost job: 7.5%, job performance suffered: 40.4%, kept job for health insurance: 30.2%, increased work hours to cover medical costs: 5.1%.	-
Tebbi et al., 1989 [44]	Employment status, job-related questions, experience in the work environment	Employment status: 62.5% of AYA cancer survivors were full-time employed, 10% part-time employed, and 27.5% unemployed. Job-related questions: 5% of AYA cancer survivors changed jobs as part of the adjustment to cancer. Experience in the work environment: 79% of AYA cancer survivors believed that readjustment to the job would be easier for survivors if the attitudes of others were changed, 64% of AYA cancer survivors believed that changes in certain physical features of the workplace were necessary to facilitate such readjustment, and 16% of AYACS believed that no changes in the workplace were necessary. AYA cancer survivors vs. comparison group: Employment status: full-time employed (62.5% vs. 65%), part-time employed (10% vs. 17.5%), unemployed (27.5% vs. 17.5%), *p* = 0.422 Job-related questions: No significant difference in experience of discrimination in hiring or promotion or problems performing their job or using job-related facilities.	-
Yanez et al., 2013 [46]	Employment status, cancer-related education or work interruption	Employment status: employed: 69%, homemaker: 11.5%, unemployed: 10.7%, student: 6.2% Cancer-related education/work interruption: 62.3% of AYA cancer survivors reported an interruption in education or work.	Time since diagnosis: AYA cancer survivors 25–60 months since diagnosis vs. 13–24 months since diagnosis vs. 0–12 months since diagnosis. Employment status: employed (% vs. 77.5 vs. 64.2), homemaker (11.5% vs. 9.8% vs. 9.2%), unemployed (10.7% vs. 3.8% vs. 15.8%), student (6.2% vs. 7.8% vs. 9.2%), cancer-related education/work interruption: 62.3% vs. 56.1% vs. 66.1%

Abbreviations: m, male; f, female; OR, odds ratio; CI, confidence interval; *p*, *p*-value; r, correlation coefficient; WAI, work ability index; aOR, adjusted odds ratio; CI, confidence interval; AYA, adolescent and young adult; HCT, allogeneic hematopoietic cell transplantation.

**Table 5:** Impact of cancer on financial outcomes in adolescent and young adult (AYA) cancer survivors.

First Author, Publication Year	Measurements for Financial Outcomes	Financial Outcomes	Determinants for Adverse Financial Outcomes (Quantitative Studies) or Selected Citations (Qualitative Studies, Indicated in Italics)
Abdelhadi et al., 2021 [23]	Annual medical expenses	AYA cancer survivors without chronic conditions had an average of USD 5468 (95% CI, USD 3128 to USD 9559) in annual medical expenditures.	Chronic conditions: AYA cancer survivors with at least one chronic condition (74% of all AYA cancer survivors) spent an additional USD 2777 (95% CI: USD 480 to USD 5958) annually compared to survivors without chronic conditions. AYA cancer survivors with four or more chronic conditions (22%) had an increased average annual medical expenditure of USD 11,178 (95% CI: USD 6325 to USD 18,503). Higher annual medical expenses: physically inactive (USD 3558; 95% CI: USD 2200 to USD 4606), having a usual source of care (USD 687; 95% CI: USD 173 to USD 1415), having regular check-ups during the last year (USD 1117; 95% CI: USD 560 to USD 1867), unable to get care when needed (USD 1291; 95% CI: USD 198 to USD 3335)
Abdelhadi et al., 2022 [29]	Annual medical expenditures	AYA cancer survivors vs. comparison group: AYA cancer survivors without psychological distress had an average of USD 5324 (95% CI, USD 3275–USD 8653) in annual medical expenditures; adults with no history of cancer without psychological distress had an average of USD 2527.03 (USD 1837.76–USD 3474.83) in annual medical expenditures.	Psychological distress: AYA cancer survivors with psychological distress had significantly higher medical expenditures than AYA cancer survivors without psychological distress (*p* for interaction = 0.013) AYA cancer survivors vs. comparison group: In AYA cancer survivors, psychological distress was associated with an additional USD 4415 (95% CI, USD 993–USD 9690) in annual medical expenditures (*p* = 0.006), In matched adults without a history of cancer, psychological distress was associated with an additional USD 1802 (95% CI, USD 440–USD 3791) in annual medical expenditures (*p* = 0.005)
Drake et al., 2019 [49]	Perspectives on and experiences with return to work following treatment	Identified themes: uncertainty about return to work, cancer as a catalyst for a career change, importance of employment benefits, benefit of YA-specific resources	*“(…) so, part of the challenge is as much as I want a new job, umm I know that my cancer is now a pre-existing condition. So, if I was to switch to a different employer, some things won’t be covered anymore. So, part of me thinks I can’t leave my job because I’m covered under my benefits now and if I was to get new benefits then this is a pre-existing condition that won’t be covered.”*
Ekwueme et al., 2016 [32]	Income, indirect productivity costs	Income: low (< USD 34,999) 30.59%, medium (USD 35,000–USD 74,999) 29.08%, high (> USD 75,000) 28.59% Indirect productivity costs: AYA cancer survivors missed 19 work days and 17 home productivity days. This resulted in indirect productivity costs of USD 2293 for missed work and USD 442 for missed home productivity days per capita per year. AYA cancer survivors vs. women aged 18–44 without breast cancer: Income: Low (< USD 34,999) 30.59% vs. 33.54%, medium (UDS 35,000–USD 74,999) 29.08% vs. 29.69%, high (> USD 75,000) 28.59% vs. 24.11% Indirect productivity costs: AYA cancer survivors had higher indirect productivity costs (from work days lost and home productivity days lost) per capita.	-
Ghaderi et al., 2013 [33]	Attendance benefit, basic benefit, medical rehabilitation benefit, disability pension	Uptake of benefits (childhood (0–14 years old at diagnosis) vs. AYA (15–19 and 20–24 years old at diagnosis) survivors): Attendance benefit: 20.5% vs. 3.3% and 1.9%, basic benefit: 19.12% vs. 8.05% and 5.12%, medical rehabilitation benefit: 9.18% vs. 10.9% and 10.3%, disability pension: 11.36% vs. 6.9% and 6.6%	Age at diagnosis: uptake of benefits (15–19 vs. 20–24 years at diagnosis): attendance benefit: 3.3% vs. 1.9%, basic benefit: 8.05% vs. 5.12%, medical rehabilitation benefit: 10.9% vs. 10.3%, disability pension: 6.9% vs. 6.6%
Ghazal et al., 2021 [51]	Perspectives of work-related goals	Identified themes: self-identity and work, perceived health and work ability, financial toxicity	*“I ended up getting into some credit card debt. I sold a lot of things that I had bought for myself over the years to try to play catch up on bills that I had monthly.”* *“I feel like I need to go do these [new WRGs], but there’s that whole financial portion.”*
Gupta et al., 2020 [52]	Experience of cancer-related financial stress	Identified themes: managing health care costs with limited funds, limiting future possibilities of employment and education, developing independence while being financially dependent, potential benefit of financial stress, work environment	*“One thing I would advise [...] is to make sure to have health insurance. [...] You know, most young adults don’t think [about] having it. “Nothing’s going to happen to me. Why do I need health insurance?” (Male, 24 years old)*
Guy et al., 2014 [34]	Family income, direct medical costs, indirect medical costs	Family income: 21.4% of AYA cancer survivors had a low family income, 41.6% had a middle family income, and 12.3% had a high family income. Annual direct medical costs: AYA cancer survivors had annual per person medical expenditures of USD 7417. Private insurance was the largest source of payment for AYA cancer survivors (USD 3083). Ambulatory and inpatient care were the largest type of service for AYA cancer survivors (USD 2409 + USD 1605). Annual indirect medical costs: All types of lost productivity resulted in a total per capita spending of USD 4564. AYA cancer survivors vs. comparison group: family income: low, 21.4% vs. 16.7%; middle, 41.6% vs. 44%; high, 12.3% vs. 16.3% Annual direct medical costs: Annual per person medical expenditures were USD 7417 vs. $4247. Private insurance was the largest source of payment, USD 3083 vs. USD 1825. Ambulatory and inpatient care saw the largest share of medical expenditures, USD 2409 + USD 1605 vs. USD 1376 + USD 1169 Annual indirect medical costs: AYA cancer survivors reported higher productivity costs due to employment disability, more missed work days among employed people, and greater household productivity loss. All types of lost productivity resulted in a higher total per capita spending of USD 4564 vs. USD 2314.	-
Kent et al., 2012 [53]	Perspectives on cancer survivorship	Concerns about being un- or underinsured as an AYA cancer survivor because they could not afford coverage and/or felt they did not need coverage. About 1/3 of survivors reported difficulties with acquiring or maintaining health insurance. Insured patients were worried about future insurability. Many survivors experienced a gap in coverage between high school, college, and full-time employment. As a result, many survivors first sought out the emergency room due to lack of insurance. Eventually, many uninsured survivors were able to obtain government-sponsored insurance, but in all cases, they indicated that this process delayed their treatment.	*“I was going to the doctors. And I was paying cash. We didn’t have insurance at that time. And when they found out from the labs that I had cancer, I went to the emergency room because I was almost dying.” (Female, diagnosed with non-Hodgkin lymphoma in her midtwenties)*
Ketterl et al., 2019 [24]	Borrowing money or going into debt	14.4% reported that they borrowed ≥ USD 10,000. 1.5% reported that they had filed for bankruptcy because of their cancer.	Treatment: Chemotherapy: inference with job-related physical tasks (OR = 1.97, CI = 1.22 to 3.11, *p* < 0.01), inference with mental tasks required by a job (OR = 3.22, CI, 2.15 to 4.79, *p* < 0.01), time off from work (OR = 3.56, CI = 2.31 to 5.47, *p* < 0.01), borrowing ≥ USD 10,000 (OR = 3.05, CI = 1.53 to 6.09, *p* < 0.01) compared with survivors who were not exposed to chemotherapy. Radiation: interference with job-related physical tasks (OR = 1.66, CI = 1.08 to 2.41, *p* < 0.05) compared with survivors who did not receive radiation. Surgery: extended paid time off from work (OR = 0.54, CI = 0.54 to 1.00, *p* < 0.05) compared with survivors who did not receive surgery.
Landwehr et al., 2016 [36]	Use of a funding grant, net worth (value of all things owned by an individual), out-of-pocket medical expenses, financial indices	Use of a funding grant: medical/insurance (34%), rent/mortgage (25%), health/wellness (20%), continuing education/loans (14%), car-related (12%), computer (10%), family building (7%), other (12%). AYA cancer survivors vs. comparison group: Net worth: AYA cancer survivors had an average negative net worth value of −USD 35,009.41 in debt compared to young adults from the general population who had a mean net worth of USD 68,479 in assets. Out-of- pocket medical expenses: AYA cancer survivors had higher expenses (mean = USD 2528.76 annually) compared to young adults from the general population (median = USD 610.00 annually).	Age at application (19–29 years old vs. 30–39 years old): Financial indices: mean total liabilities: USD 37,760.16 vs. USD 59,012.16 (*p* < 0.05), mean total medical debt: USD 3616.89 vs. USD 4239.34, mean total credit card debt: USD 3025.93 vs. USD 3913.89, mean monthly income: USD 1385.84 vs. USD 1851.14 (*p* < 0.05), mean monthly expenses: USD 1490.94 vs. USD 2135.70 (*p* < 0.01), mean monthly medical expenses: USD 184.25 vs. USD 242.82, mean monthly student loan payment: USD 112.35 vs. USD 68.53, mean income to expenses ratio: 0.87 vs. 0.89
Lu et al., 2021 [38]	Medical financial hardship	The majority of AYA cancer survivors (62.2%) experienced at least one domain of medical financial hardship. Material hardship (reporting problem paying medical bills): 36.7%, psychological hardship (reporting worry about medical costs): 46.6%, behavioral hardship (reporting delaying or forgoing medical care because of worry about cost or being unable to afford prescription medicine or care): 28.4%. AYA cancer survivors vs. comparison group: AYA cancer survivors were significantly more likely to experience medical financial hardship compared to adults without a cancer history. Material hardship (36.7% vs. 27.7%, *p* < 0.001), psychological hardship (46.6% vs. 44.7%, *p* = 0.210), behavioral hardship (28.4% vs. 21.2%, *p* < 0.001).	-
Meernik et al., 2020 [25]	Financial hardship	Financial hardship: 27% of AYA cancer survivors reported financial hardship (borrowing money, going into debt, and/or filing for bankruptcy), 27% had borrowed money or gone into debt, and 3% reported to have filed for bankruptcy.	Employment disruption: Financial hardship differed significantly between AYA cancer survivors with and without employment disruption: 43% vs. 20%, borrowing money or going into debt: 43% vs. 20%, filing for bankruptcy: 4% vs. 2%.
Nord et al., 2015 [39]	Disability pension	AYA cancer survivors vs. comparison group: number of persons with disability pension: 76/2073 (4%) vs. 209/8140 (3%).	Disability pension: Extensive treatment with 4 courses (HR = 1.93, CI = 1.01 to 3.71), extensive treatment with ≥ 4 courses (HR = 5.16, CI = 2.00 to 10.3)
Tangka et al., 2020 [43]	Treatment and other non-clinical costs, financial decline	Treatment and other non-clinical costs: 27.7% of women spent less than USD 500, 27.9% spent USD 500 to USD 2000, 18.7% spent USD 2001 to USD 5000, and 17.0% spent USD 5001 to USD 10,000 out of pocket for breast cancer treatment (e.g., for hospital bills, deductibles, and medication) during the 12 months prior to the study. For these costs, most women used personal funds (81.5%), informal borrowing from family and friends (22.9%), the method of leaving some medical bills unpaid (22.7%), or increasing credit card debt (21.7%). Financial decline: 47.0% of women experienced a financial decline due to their cancer diagnosis.	Women showing the following characteristics were most vulnerable to financial decline due to their cancer diagnosis: non-Hispanic other: OR = 2.58 (compared to non-Hispanic White women), some college education: OR = 1.58 (compared to women with a college or postgraduate degree), one comorbidity: OR = 1.80 (compared to women with no comorbid conditions), two or more comorbidities: OR = 2.80 (compared to women with no comorbid conditions), late-stage diagnoses (stage III and IV): OR = 1.76 (compared to women diagnosed at earlier stages), self-funded insurance: OR = 2.29 (compared to women with employer-based insurance coverage).
Tebbi et al., 1989 [44]	Income	Income: AYA cancer survivors had a mean income of USD 16,750. AYA cancer survivors vs. comparison group: mean income: USD 16,750 vs. USD 12,250, *p* = 0.006	-
Thom et al., 2021 [45]	Financial toxicity, medical cost-coping	Financial toxicity: The mean score for financial toxicity was 14.0 (±9.33), which indicates severe financial toxicity in AYA cancer survivors. Medical cost-coping: Participants on average reported 3.2 (± 1.89) cost-coping behaviors, including postponing mental health care (46% of the sample) and/or preventative care (36%); having a health problem but not seeing a provider (37%); skipping a medical test, treatment, or follow-up (34%); and not filling a prescription (27%) or taking a smaller dose of a medication than prescribed (18%).	Financial toxicity was associated with: full-time employment (mean difference of the financial toxicity score between people lacking and people having full-time employment: −4.66; 95% CI: −7.18 to −2.13), less education (correlation coefficient r = 0.31; *p* < 0.001), lower income (r = 0.47; *p* = < 0.001), younger age at time of survey completion (r = 0.16; *p* = 0.05), more COVID-19 pandemic-related negative economic events (e.g., not having enough money for medical expenses, food or medication) (r = −0.59; *p* = < 0.001).

Abbreviations: OR, odds ratio; CI, confidence interval; *p*, *p*-value; WRG, work-related goal; AYA, adolescent and young adult; r, correlation coefficient.

## Supplementary Materials

The following supporting information can be downloaded at: https://www.mdpi.com/article/10.3390/curroncol30100631/s1, Table S1: Characteristics of included quantitative studies (detailed version); Table S2: Characteristics of included qualitative studies (detailed version); Table S3: PICO format for the research questions; Table S4: Search blocks for the search in the literature databases; Table S5: Quality assessment for quantitative cross-sectional studies; Table S6: Quality assessment for qualitative studies.
