# Supplementary material for: Education, Employment, and Financial Outcomes in Adolescent and Young Adult Cancer Survivors—A Systematic Review
Source: Curr Oncol. 2023 Sep 25;30(10):8720–62. doi: 10.3390/curroncol30100631 (PMC10604989; doi:10.3390/curroncol30100631)
Supplement: Supplementary file 1 [file curroncol-30-00631-s001.zip › curroncol-2520571-supplementary.pdf]

Supplementary Material

Education, Employment, and Financial Outcomes in Adolescent and Young Adult Cancer Survivors—A Systematic Review

Aurelia Altherr [1], Céline Bolliger [1], Michaela Kaufmann [1], Daniela Dyntar [1,2], Katrin Scheinemann [1,3,4], Gisela Michel [1], Luzius Mader [5,6] and Katharina Roser [1,*]

1 Faculty of Health Sciences and Medicine, University of Lucerne, Alpenquai 4, 6005 Lucerne, Switzerland
2 Cancer Registry of Central Switzerland, 6000 Lucerne, Switzerland
3 Division of hematology & oncology, Children's Hospital of Eastern Switzerland, 9006 St. Gallen, Switzerland
4 Department of Pediatrics, McMaster Children's Hospital and McMaster University, Hamilton, L8N 3Z5 ON, Canada
5 Institute of Social and Preventive Medicine, University of Bern, 3012 Bern, Switzerland
6 Cancer Registry Bern-Solothurn, University of Bern, 3008 Bern, Switzerland
* corresponding author

Corresponding author:
Katharina Roser, Faculty of Health Sciences and Medicine, University of Lucerne, Alpenquai 4, 6005 Lucerne, Switzerland; katharina.roser@unilu.ch

Content

Table S1: Characteristics of included quantitative studies (detailed version)..........2
Table S2: Characteristics of included qualitative studies (detailed version). 13
Table S3: PICO formats for the research questions. 16
Table S4: Search blocks for the search in the literature databases. 17
Table S5: Quality assessment for quantitative cross-sectional studies. 18
Table S6: Quality assessment for qualitative studies. 19

Table S1: Characteristics of included quantitative studies (detailed version).

First author, publication year	Country	Study design	Data source	Data collection method	Sample size	Response rate	Gender: percentage male	Age at time of study	Age at diagnosis	Time since diagnosis	Education, employment, and/or financial outcomes (not main outcomes, additionally reported)	Cancer types	Comparisons
Abdelhadi 2021 et al. [23]	USA	Retrospective cohort study	2011-2016 Medical Expenditure Panel Survey (MEPS)	in-person interviews and questionnaire	n = 2326	MEPS (2011-2016): 53.5-59.3% for the different years	AYA cancer survivors with chronic conditions: 23.90% male, AYA cancer survivors without chronic conditions: 21.85% male	(weighted proportions)<br>AYA cancer survivors with chronic conditions:<br>18-29 years: 6.14%, 30-39 years: 15.52%, 40-49 years: 24.36%, 50-64 years: 36.10%, ≥65 years: 17.88%<br>AYA cancer survivors without chronic conditions:<br>18-29 years: 18.14%, 30-39 years: 37.52%, 40-49 years: 27.82%, 50-64 years: 13.90%, ≥65 years: 2.70%	range: 15-39 years	AYA cancer survivors with chronic conditions:<br>0-4 years: 10.86%, 5-9 years: 12.73%, 10-19 years: 26.31%, ≥20 years: 50.09%, AYA cancer survivors without chronic conditions:<br>0-4 years: 31.85%, 5-9 years: 22.96%, 10-19 years: 29.43%, ≥20 years: 15.76%	(weighted proportions)<br>**Education (s)**:<br>AYA cancer survivors with chronic conditions:<br>less than high school: 38.10%, high school graduate: 35.00%, some college or more: 26.90%, AYA cancer survivors without chronic conditions:<br>less than high school: 35.03%, high school graduate: 26.71%, some college or more: 38.26%<br>**Income (s)**:<br>AYA cancer survivors with chronic conditions:<br>poor: 15.97%, near poor: 6.30%, low: 14.41%, middle: 25.52%, high: 37.80%	(weighted proportions)<br>AYA cancer survivors with chronic conditions:<br>bladder: 0.70%, brain: 1.69%, breast: 12.57%, cervix: 32.90%, colon: 2.94%, leukaemia: 1.72%, lung: 2.07%, lymphoma: 4.42%, melanoma: 9.26%, other: 28.26%, prostate: 1.70%, throat: n/a, thyroid: 3.90%<br>AYA cancer survivors without chronic conditions:<br>bladder: n/a, brain: n/a, breast: 11.15%, cervix: 21.86%, colon: 1.76%, leukaemia: 1.52%, lung: n/a, lymphoma: 5.45%, melanoma: 10.94%, other: 26.55%, prostate: n/a, throat: n/a, thyroid: 8.50%	none

											AYA cancer survivors without chronic conditions: poor: 13.22%, near poor: 3.27%, low: 10.52%, middle: 32.95%, high: 40.04%		
Abdelhadi 2022 et al. [29]	USA	Retrospective cohort study	2011-2016 Medical Expenditure Panel Survey (MEPS)	in-person interviews and questionnaire	n = 2081 (n = 1757 for matched analyses)	MEPS (2011-2016): 53.5-59.3% for the different years	20.0% male	18-29 years: 10.2%, 30-39 years: 22.9%, 40-49 years: 27.3%, 50-64 years: 26.6%, ≥65 years: 13.0%	range: 15-39 years	not reported	**Education (s):** less than high school: 27.1%, high school graduate:18.9%, some college or more: 13.5%	not reported	adults without cancer history (n = 5227)
Bhatt 2021 et al. [30]	USA	Retrospective cohort study	Center for International Blood and Marrow Transplant Research (CIBMTR)	standard data collection forms of CIBMTR	n = 1365	not applicable	56% male	not reported	mean age at treatment =30.8 years, range=18-39 years, 18-24 years: 19%, 25-29 years: 26%, 30-34 years: 27%, 35-39 years: 28%	not reported	**Highest education (d):** high school or less: 36%, college: 15%, graduate school: 26%, missing: 22% **Employment status (d):** full-time: 43%, part-time: 4%, unemployed: 19%, medical disability: 16%, unknown: 17%	leukaemia: 68%, lymphoma: 11%, other diseases apart from cancer: 21%	none
Dahl 2019 et al. [31]	Norway	Cross-sectional study	Cancer Registry of Norway	self-reported information	n = 1189	42%	27% male	mean (SD)=49.7 (7.8), median = 49 years, range: 27-65 years	mean (SD)=33.0 (5.3), median = 35 years range: 19-39 years,	median =16 years, range: 6-31 years	**Educational attainment (s):** high (>12 years): 59%, low (<12 years): 41% **Employment status (s):** employed: 75%, non-employed: 25%	breast: 41%, colorectal: 12%, lymphoma: 19%, leukaemia: 11%, melanoma: 17%	none

Dieluweit 2011 et al. [20]	Germany	Cross-sectional study	German Childhood Cancer Registry	self-reported information	n = 820	43.70%	49% male	mean (SD)=29.9 (6) years	mean (SD)=15.8 (0.9) years, range: 15-18 years,	mean (SD)=13.7 (6) years	**University entrance qualification (s):** 52.4% of survivors, 38.3% of comparisons<br>**Employed (s):** 79.6% in survivors, 74.2% in comparisons	lymphoma: 30.5%, malignant bone tumour: 21.2%, leukaemia: 19.3%, CNS tumours: 9.5%, soft tissue and other extraosseous sarcomas: 9.2%, germ cell tumours: 6.6%, other malignant epithelial neoplasms and malignant melanomas: 2.4%, renal tumours: 0.9%, neuroblastoma: 0.5%	age-matched sample from the general population (German Socio- Economic Panel, n = 820)
Ekwueme 2016 et al. [32]	USA	Cross-sectional study	CDC's National Center for Health Statistics	National Health Interview Survey (NHIS), a cross-sectional survey	n = 244	not reported	all female	mean (SD)=39.42 (5.29) years	mean (SD)=34.42(6.95) years, range: 18-44 years,	<2 years: 30.74%, 2-4 years: 28.69%, 5-10 years: 29.1%, ≥11 years: 11.48%	women aged 18-44 with breast cancer vs women aged 18-44 without breast cancer vs women aged 44-64 with breast cancer:<br>**Employment (s):** employed: 75.43% vs 78.38% vs 64.61%<br>**Income (s):** low (<34,999$): 30.59% vs 33.54% vs 25.88%, medium (35,000$-74,999$): 29.08% vs 29.69% vs 25.93%, high (>75,000$): 28.59% vs 24.11% vs 16.4%, bachelor/master degree: 29.46% vs 26.67% vs 32.33	all breast	women aged 18-44 years without breast cancer (n = 82694), women aged 45-64 at diagnosis with breast cancer (n =1508), women aged 45-64 years without breast cancer (n =52586)

Ghaderi 2013 et al. [33]	Norway	Retrospective cohort study	Cancer Registry of Norway	not reported	n = 2561	not reported	55.4% male (childhood and AYA cancer survivors)	not reported	15-19 years: 1019, 20-24 years: 1542, mean follow-up time of study of 5-year survivors=13.2 years range: 0-39.3 years (childhood and AYA cancer survivors)	not reported	not reported	brain/CNS tumours: 18.2%, testis: 15.4%, lymphatic system: 14.4%, hematopoietic system: 12.9%, melanoma: 10.6%, other: 7.4%, thyroid gland and other endocrine glands: 7.3%, bone and connective tissue: 5.6%, kidney: 2.7%, eye: 2.2%, ovary: 2%, cervix uteri: 1.2% (childhood and AYA cancer survivors)	childhood cancer survivors (0-14 years of age at diagnosis; n = 1470)
Guy 2014 et al. [34]	USA	Retrospective cohort study	Medical Expenditure Panel Surveys (MEPS) 2008-2011	not reported	n = 1464	MEPS (2008-2011: 53.5-59.3%	22.2% male	18-29 years: 11%, 30-39 years: 21%, 40-49 years: 26.7%, 50-64 years: 29.3%, ≥65 years: 12%	range: 15-39 years	0-9 years: 30.5%, 10-19 years: 27.7%, ≥20 years: 41.9%	survivors vs comparisons: **Educational attainment (s):** less than high school graduate: 15.5% vs 16.9%), high school graduate: 29.9% vs 29.5%, some college or more: 54.7% vs 53.2%, **Family income (s):** low: 21.4% vs 16.7%, middle: 41.6% vs 44%, high: 37.0% vs 39.3%	not reported	adults without cancer in the pooled sample of 2008-11 MEPS data (n = 86865)
Hamzah 2021 et al. [35]	Malaysia	Cross-sectional study	National Cancer Institute of Malaysia and Kuala Lumpur General Hospital	hospital interviews	n = 400	not reported	43.3% male	mean (SD)=29.1 (7.16) years, range: 18-40, 18-20 years: 12.5%, 21-25	not reported	>5 years	**Educational attainment (s):** PhD: 0.5%, master degree: 5.3%, bachelor degree: 31.8%, diploma: 31.5%,	leukaemia: 32.25%, Hodgkin lymphoma: 10.0%, ovarian: 8.0%, ependymoma: 7.25%, breast: 6.25%,	none

								years: 27%, 26-30 years: 17.8%, 31-35 years: 12.8%, 36-40 years: 30%			higher school certificate: 4.8%, certificate of education: 23.5%, certificate of lower education: 2.9% **Employment (s):** permanent employment: 67.5%, temporary employment: 12.5%, self-employed: 14.8%, part-time employed: 5.2% **Monthly income (s):** <RM3,00: 49.5%, RM3,001-RM5,000: 34.0%, RM5,001-RM10,00: 13.3%, >RM10,001: 0.5%	Wilms' tumour: 5.75%, Ewing's sarcoma: 5.75%, testicular: 3.5%, medulloblastoma: 3.5%, brain tumour: 3.25%, yolk sac tumour: 3%, liver cancer: 2.75%, papillary thyroid: 1.5%, nasopharyngeal cancer: 1.5%, neuroblastoma: 1.5%, intestinal: 1.25%, lung: 1%, germinoma: 1%, embryonal rhabdomyosarcoma: 1%	
Ketterl 2019 et al. [24]	USA	Cross-sectional study	collected data	online survey	n = 872	67%	27.2% male	not reported	females: mean (SD)=32.3 (5.62) years, males: mean (SD)= 29.8 (6.09) years,	females: mean (SD) =3.53 (1.49) years, males: mean (SD)=3.40 (1.29) years	**Educational attainment (s):** high school or less: 6.9%, some college: 22%, college graduate: 38%, post graduate: 33.1%	breast: 27.6%, leukaemia and lymphoma: 18.7%, endocrine system: 14.7%, skin: 9.3%, genital system: 10.9%, brain and other CNS tumours: 4.7%, bones and soft tissue: 4.1%, digestive system: 4.0%, oral cavity and pharynx: 2.9%, urinary system: 1.6%, others: 1.5%	none
Landwehr 2016 et al. [36]	USA	Retrospective cohort study	information on grant applications submitted between 2007	not reported	n = 334	33.60%	20.4% male	age at time of application submission: mean =29.3 years, median	mean (SD)=24.5 (6.7) years, median = 26 years, 95% CI: [23.7-25.2],	time of treatment completion prior application submission:	not reported	not reported	US census data from 2011 and 2013, using the groups

			and 2013 to Samfund, which is a national non-profit organization that helps AYACS reduce debt and live independently.					= 30.0 years, 95% CI: 28.7-29.8, SD=4.4 years, range: 19-39 years		mean (SD)=3.5 (4.6) years, median = 1.8 years, 95% CI: 3.0-4.0			“under age 35” and “25–34 years of age”, n = 16513000, and MEPS, using the group “18–44 years of age”, n = 21877000
Lim 2020 et al. [37]	Switzerland	Retrospective cohort study	institutional database from Paul Scherrer Institute (PSI)	not reported	n = 176	not applicable	43.2% male	not reported	median (SD) age at treatment =30.3 (±7.6) years, range: 15.1-39.5 years,	median time since treatment=66 months, range: 12-236 months	for 63 participants with longitudinal data available: **Employment (t):** unemployed: 9.5%, employed (100% sick leave): 14.3%, employed (at work): 42.9%, in education: 33.3% **Employment (fu):** unemployed: 23.8%, employed (at work): 63.5%, in education: 12.7%	all brain and skull base tumours	none
Lu 2021 et al. [38]	USA	Cross-sectional study	2010-2018 National Health Interview Surveys (NHIS)	in-person interviews	n = 2588	NHIS (2010-2018)64.2-82.0% for the different years	32.8% male	18-29 years: 8.3%, 30-39 years: 23.0%, 40-49 years: 26.1%, 50-64 years: 27.4%, 65-80 years: 12.2%, 81+ years: 2.9%	Median (IQR)=31 (26-35) years	(categories are not mutually exclusive), <2 years: 8.4%, ≥2 years: 91.6%, >6 years: 75%, >16 years: 50%, >31 years: 25.0%	**Education (s)**: <high school or missing: 10.5%, high school: 22.5%, ≥some college: 67% **Family income (s)** (as % of federal poverty level): <200: 26.6%, ≥200 and <400: 25.7%, ≥ 400: 40.6%, missing: 7.1%	lymphoma: 7.8% , melanoma: 12.3%, testicular cancer: 5.5%, thyroid cancer: 9.1%, ovarian cancer: 7.3%, uterine cancer: 10.8%, leukaemia: 1.9%, breast cancer: 15.7%	adults without cancer history (n = 256964)
Mader 2017 et al. [19]	Switzerland	Cross-sectional study	Cancer Registry Zurich and Zug and	self-reported information	n = 160	41.10%	61.3% male	mean (SD)=33.5(5.9) years, 20-29	mean (SD)=21.1 (2.9) years, range: 16-25	mean (SD)=11.9 (4.7) years	survivors vs comparisons:	lymphoma: 37.5%, germ cell tumour: 28.8%, CNS tumour:	Swiss Health Survey

								years: 26.9%, 30-29 years: 53.1%, ≥40 years: 20%	years, 16-20 years: 43.8%, 21-25 years: 56.3%,		**Educational achievement (s):** basic: 8.2% vs 4.8%, vocational training: 46.5% vs 47.2%, upper secondary: 33.3% vs 26.7%, university: 11.9% vs 21.3% **Employment status (s):** employed: 91.2% vs 89.5%, unemployed: 8.8% vs 10.5%	9.4%, soft tissue sarcoma: 9.4%, leukaemia: 8.1%, bone tumour: 3.8%, renal tumour: 1.9%, neuroblastoma: 1.3%	(SHS), participants aged 20–50 years resident in the Canton of Zurich (n = 999)
Meernik 2020 et al. [25]	USA	Cross-sectional study (restricted to working (full/part-time) at time of diagnosis)	information from the North Carolina Central Cancer Registry and the Kaiser Permanente Northern California (KPNC) and Southern California (KPSC) tumour registries	online survey	n = 1328	12.80%	all female	median (SD)=41.0 (6.2) years	median (SD)=34.0 (5.1) years, range: 16-39 years	median (SD)=7.0 (3.6) years, range: 3-15 years	**Employment status (d):** part-time: 17%, full-time: 82.6%, employed but unknow status: 0.4% **Educational attainment (s):** high school or less: 4.7%, some college: 29.4%, bachelor degree or more: 65.9%	breast: 41.7%, thyroid: 22.3%, melanoma: 14.4%, lymphoma: 10.4%, gynaecologic (cervical, uterine, ovarian): 11.2%	none
Nord 2015 et al. [39]	Sweden	Retrospective cohort study	SWE-NOTECA (Swedish Norwegian Testicular Cancer Group) database	not reported	n = 2146	not reported	all male	not reported	median =32 years, range: 18-60 years	follow-up for study: median = 10 years, range: 2-19 years	**Education (s):** ≤9 years: 15%, 10-12 years: 54%, >12 years: 30%	all testicular	general population without a cancer history (n = 8448)
Nugent 2018 et al. [40]	USA	Cross-sectional study	collected data	self-reported information	n = 23	not reported	69.9% male	mean (SD)=23.8 (4.0) years, median	mean = 17.4 years, range: 15-21 years, length	≥2 years since active cancer treatment	**Duration of education (s):**	Hodgkin lymphoma: 43.4%, acute	controls were matched to

								(IQR)=22.6 (5.0)	of treatment: mean =1.2 years		mean (SD)=14.7(2.4) years, median (IQR)= 15.0 (5.0) years	lymphoblastic leukaemia:17.4%, Ewing's sarcoma: 8.7%, osteosarcoma: 8.7%, germ cell tumour: 8.7%, acute myelocytic leukaemia: 4.3%, chondrosarcoma: 4.3%, non-Hodgkin lymphoma: 4.3%	the cancer survivors, being of the same gender and within 2 years of the survivor's age (n = 14)
Parsons 2012 et al. [17]	USA	Cohort study	AYA Hope Study, observational cohort study	self-report survey	n = 463 (all AYA cancer survivors)	Initial survey: 43.4%, follow-up survey: 88.7%	AYA cancer survivors working or in school full-time before diagnosis (n = 388):, 64% male	not reported	AYA cancer survivors working or in school full-time before diagnosis (n = 388):, 15-19 years: 13.1%, 20-24 years: 17.8%, 25-29 years: 24.7%, 30-34 years: 23.2%, 35-39 years: 21.1%	AYA cancer survivors working or in school full-time before diagnosis (n = 388):, 15-19 months: 13.1%, 20-24 months: 42.5%, 25-29 months: 34%, 30-35 months: 10.1%, range: 25-35 months	**Employment (d):** unemployed: 4.3%, homemaker: 3.0%, part-time: 8.9%, full-time: 83.8%	germ cell: 40.5%, Hodgkin's lymphoma: 26%, non-Hodgkin's lymphoma: 24.2%, sarcoma: 4.6%, acute lymphoblastic leukaemia: 3.9%	AYA cancer survivors 15-24 months after diagnosis and working or in school full-time before diagnosis (n = 216)
Strauser 2010 et al. [41]	USA	Longitudinal study (restricted to AYACS who were unemployed at time of application for vocational services)	U.S. Department of Education, Rehabilitation Service Administration (RSA) Case Service Report (Form 911) database	not reported	n = 368	not reported	57% male	mean (SD)=21.46 (2.39) year, range: 18-25 years	not reported	>2 years	**Education (s):** special education: 6%, less than high school: 33.4%, completed high school: 42.7%, post-secondary/associate: 16.6%, college degree or higher: 1.4%	not reported	none
Sylvest 2022 et al. [42]	Denmark	Register-based cohort study	Danish Cancer Register, several Danish population registers	not reported	n = 4222	not applicable	100% male	≥35 years	range: 0-29 years,	CNS cancer: mean (SD)=14.59 (9.30) years,	For age group 20-29 years at diagnosis **Education (s):**	CNS tumours: 5.0%, haematological tumours: 6.5%, solid tumours: 88.5%	age-matched comparison group of the

										haematological cancer: mean (SD)=16.68 (10.67) years, solid cancer: mean (SD)=9.37 (8.47) years	primary school: 19%, high school: 6%, vocational training: 40%, short further education: 6%, medium length further education: 15%, long further education: 15% **Employment (s):** employed: 84%, unemployed but in the workforce: 3%, outside the workforce (retired/receiving transfer income): 8%, other: 4%		general population (n = 794589)
Tangka 2020 et al. [43]	USA	Cross-sectional study	state cancer registries in California, Florida, Georgia, and North Carolina + data collected for the study	paper and online questionnaire	n = 830	28.40%	all female	not reported	18-34 years: 39.5%, 35-39 years: 60.5%	not reported	**Education (s):** graduate degree: 23.1%, bachelor's degree: 34.3%, some college: 27.2%, high school or less: 13.6%, missing: 1.9% **Employment (d):** employed: 73.4%	all breast cancer	none
Tebbi 1989 et al. [44]	USA	Cross-sectional study	collected data	survey	n = 40	30%	40% male	mean (SD)=26.4 (4.2) years, range: 18-35 years	mean = 16.15 years, range: 13-19 years	mean (SD)=10.1(3.2) years	survivors vs comparisons: **Employment status (s):** unemployed: 27.5% vs 17.5%, part-time employed: 10.0% vs 17.5%, full-time employed: 12.5% vs 17.5%	Hodgkin's/non-Hodgkin's lymphoma: 47.5%, soft tissue sarcoma/melanomas: 20.0%, leukaemia: 7.5%, bone tumours: 20.0%, ovarian/testicular: 5.0%	15 male and 25 female controls without a cancer history and with age range from 18 to 35 years (n = 40)

Thom 2021 et al. [45]	USA	Cross-sectional study	collected data for the study	online questionnaire	n = 212	65%	8.9% male	mean (SD)=35.3 (5.25) years	mean (SD)=27.4 (7.17) years	mean (SD) time since treatment=6.2 (5.89) years	**Highest education (s)**: high school: 3.8%, some college or vocational training: 11.3%, associate's degree: 8.0%, bachelor's degree: 32.5%, graduate or professional degree: 42.0%, prefer not to respond: 2.4% **Employment status (s)**: working full-time: 58.0%, working part-time: 8.5%, homemaker/stay-at-home parent: 7.1%, in school: 9.0%, on disability: 17.5%, unemployed: 7.5%, prefer not to respond: 5.8% **Household income (s):** <$25,000: 13.7%, $25,000-$49,999: 17.5%, $50,000-$99,999: 35.4%, ≥$100,000: 25.9%, do not know: 2.4%, prefer not to respond: 5.2%	breast: 27.8%, lymphoma: 16.5%, colorectal: 11.3%, leukaemia: 9.4%, brain: 7.1%, gynaecological: 6.1%, sarcoma: 6.1%, thyroid: 4.7%, other: 8.0%, prefer not to respond: 0.5%	none
Yanez, 2013 et al. [46]	USA	Cross-sectional study	Collected data	online survey	n = 106	66.50%	31.6% male	mean (SD)=32.2 (5.1) years	not reported	range: 25-60 months, 3 years after treatment completion:	short-time vs intermediate vs long-term survivors: **Educational attainment (s):**	breast: 24.8%, cervical: 11.5%, melanoma: 9.7%, lung: 8.0%, colorectal:	AYA cancer survivors 0-24 months after diagnosis (n = 216)

41%, 4 years after treatment completion: 31%, 5 years after treatment completion: 28%	less than college degree: 39.2% vs 34.3% vs 41.6%<br>**Household income (s):**<br>≤74,999$: 56.7% vs 38.2% vs 55.8%<br>**Employment status (s):**<br>employed: 64.2% vs 77.5% vs 69%, homemaker: 9.2% vs 9.8% vs 11.5%, unemployed: 15.8% vs 3.8% vs 10.7%, student: 9.2% vs 7.8% vs 6.2%	3.5%, thyroid: 9.7%, testicular: 4.4%

Abbreviations: d, diagnosis; s, study; t, treatment; fu, follow-up; CI, confidence interval; IQR, interquartile range; SD, standard deviation; NHIS, National Health Interview Surveys; MEPS, Medical Expenditure Panel Survey; CNS, central nervous system; RM, Malaysian ringgit; PhD, doctoral degree

Table S2: Characteristics of included qualitative studies (detailed version).

First author, publication year	Country	Study design or approach, analysis method	Data collection method	Sample size	Gender: percentage male	Age at time of study	Age at diagnosis	Time since diagnosis	Cancer types	Education, employment, and/or finances (not main outcomes, additionally reported)
An 2019 et al. [47]	South Korea	Grounded theory/thematic analysis	in-depth interviews	n = 14	21.43% male	range: 14-22 years	not reported	not reported; adolescents who visited a hospital for follow-up care following treatment for leukaemia	acute lymphoid leukaemia: 42.9%, acute myeloid leukaemia: 50%, chronic myeloid leukaemia: 7.1%	not reported
Brauer 2017 et al. [48]	USA	Grounded theory; systematic yet flexible coding process	in-depth interviews	n = 18	61.1% male	mean = 26 years, range: 19.8-34.6 years	age at treatment: mean = 23.3 years, range: 18.5-29.7 years	time since treatment: mean =32.8 months, range: 8-60 months	acute myeloid leukaemia: 56%, acute lymphoblastic leukaemia: 28%, Hodgkin's lymphoma: 11%, non-Hodgkin's lymphoma: 5%	**Educational attainment (s):** some high school: 6%, high school degree: 11%, some college degree: 50%, 4-year college degree: 28%, graduate degree: 6% **School/employment status (s):** no school and unemployed: 33%, full-time or part-time student: 28%, full- or part-time employed: 33%, student and employed: 6%
Drake 2019 et al. [49]	Canada	Phenomenology; thematic analysis	in-depth interviews	n = 5	40% male	mean (SD)=32 (6.78) years, range: 25-40 years	range: 18-39 years	not reported	5 participants with Hodgkin's lymphoma, multiple myeloma, malignant neoplasm of the pineal region, thyroid cancer, and appendix cancer	not reported
Elsbernd 2018 et al. [50]	Denmark	Thematic analysis	semi-structured interviews	n = 9	22.2% male	mean = 24.2 years, median = 25 years, range: 19-27 years	range: 17-24 years	time since last treatment: range: <1 - >10 years	9 participants with lymphoma (2), breast (2), leukaemia, cervical, testicular, pancreatic, and brain tumour	**Educational attainment and sick-leave (s):** attending secondary or higher education: 88.9%, sick leave: 11.1%
Ghazal 2021 et al. [51]	USA	Thematic analysis combined with an abductive approach	semi-structured interview data from two interview studies: a study of health-related quality of life in testicular cancer and a study of the	n = 52	59.6% male	mean (SD)=25.29 (2.88) years, range: 18-29 years	not reported	mean (SD)=31.25 (17.12) months	hematologic: 61.5%, testicular: 38.5%	**Educational attainment (s):** college graduated: 51.9%

			psychosocial adaptation among young adults with hematologic cancer							
Kent 2012 et al. [53]	USA	Hermeneutic phenomenology (interpretative method); grounded theory; narrative analysis	focus group methodology and semi-structured interviews	n = 19	52.6% male	15-19 years: 5.3%, 20-23 years: 10.5%, 24-26 years: 15.8%, 27-29 years: 15.8%, 30-33 years: 26.3%, 34-36 years: 21.1%, 37-39 years: 5.3%	15-19 years: 15.8%, 20-23 years: 21.1%, 24-26 years: 21.1%, 27-29 years: 21.1%, 30-33 years: 10.5%, 34-36 years: 10.5%	range: 6 months-6 years	non-Hodgkin's lymphoma: 21.1%, Hodgkin's: 10.5%, brain tumour: 10.5%, acute lymphoblastic leukaemia: 10.5%, ovarian: 10.5%, melanoma: 5.3%, Wilm's tumour: 5.3%, testicular: 5.3%, ovarian: 5.3%, acute lymphoblastic leukaemia: 5.3%, multiple myeloma: 5.3%, aplastic anaemia: 5.3%	not reported
Parsons, 2008 et al. [55]	Canada	Postmodern narrative approach; data analysis occurred in conjunction with data collection	in-depth interviews	n = 14	57.1% male	mean = 27.4 years, median = 26.5 years, range: 18-38 years	mean = 24.2 years, median = 23 years, range: 16-35 years,	range: 1-6 years	all osteosarcoma	not reported
Raque-Bogdan, 2015 et al. [56]	USA	Consensual method	semi-structured interviews	n = 13	all female	range: 24-43 years	mean (SD)=30 (5) years, median = 27 years, range: 21-38 years	mean = 3.54 years	all breast	restricted to women working full-time at time of diagnosis:<br>**Educational attainment (s):**<br>college: 46%, graduate school: 54%<br>**Annual income (s):**<br><$100,000: 46%**,** ≥$100,000: 54%
Stone 2019 et al. [57]	USA	Constructivist grounded theory; analytic techniques including initial, focused, axial and theoretical coding procedures	semi-structured interviews	n = 12	25% male	mean = 43.9 years, range: 28-59 years	mean = 29 years, 18-29 years: 50% 30-39 years: 50%	mean =14.8 years, range: 8-35 years	breast: 33%, leukaemia or lymphoma: 33%, melanoma: 8%, testicular: 17%, thyroid: 8%	not reported
Ghazal 2021 et al. [51]	USA	Cross-sectional study	semi-structured interviews + questionnaire	n = 40	36.5% male	not reported	median (SD)=28 (5.26) years, range: 20-38 years	range: 1-5 years	lymphoma: 82.5%, leukaemia: 17.5%	**Employment (s)**:<br>employed: 92.5%, not employed: 2.5%, in education: 5.0%<br>**Level of education (s)**:

			survey for socio-demographic and clinical characteristics							high school: 7.5%, college: 22.5%, associate's degree: 10%, bachelor's degree: 25%, master's degree: 22.5%, professional or doctoral degree: 10%
Magrath 2021 et al. [54]	United Kingdom	Phenomenological analysis, analysis was performed iteratively	semi-structured interviews	n = 8	50% male	mean = 21.8 years, median = 21 years, range: 18-27 years	mean = 17.6 years, median = 17.5 years, range: 16-19 years	not reported	brain tumour: 12.5%, lymphoma: 75%, leukaemia: 12.5%	**Education (d)**: sixth form education: 100%, higher education: 75%

Abbreviations: d, diagnosis; s, study; t, treatment

Table S3: PICO formats for the research questions.

**Research question i): To describe education, employment, and financial outcomes in AYA cancer survivors:**	
For articles including a comparison group:	
**Population**	Adults
**Intervention/Exposure**	Survivors of cancer during adolescence or young adulthood (15 to 39 years of age)
**Comparison**	Comparison group
**Outcome**	Education, employment, and/or financial outcomes
For articles not including a comparison group:	
**Population**	Survivors of cancer during adolescence or young adulthood (15 to 39 years of age)
**Intervention/Exposure**	-
**Comparison**	-
**Outcome**	Education, employment, and/or financial outcomes
**Research question ii): To describe determinants for adverse educational, employment, and financial outcomes in AYA cancer survivors:**	
**Population**	Survivors of cancer during adolescence or young adulthood (15 to 39 years of age)
**Intervention/Exposure**	Different levels of various characteristics
**Comparison**	Different levels of various characteristics
**Outcome**	Education, employment, and/or financial outcomes

Table S4: Search blocks for the search in the literature databases.

**Block 1: Outcomes (education, employment, financial outcomes)**	(employment [MeSH terms] OR absenteeism [MeSH Terms] OR sick leave [MeSH Terms] OR rehabilitation, vocational [MeSH Terms] OR vocational guidance [MeSH Terms] OR occupation[MeSH Terms] OR occupation* OR return to work[mh] OR employment OR unemploy* OR vocational OR vocation* OR career OR income OR benefits use[tiab] OR socioeconomic status[tiab] OR insurance, disability[mh] OR disability insurance[tiab] OR salaries and fringe benefits[mh] OR salaries and fringe benefits OR social security OR financial burden OR financial toxicity OR economic burden OR financial hardship) OR (educational status[mh] OR education[mh] OR education* OR level of education)
**Block 2: Adolescents and young adults (AYAs)**	("young adult" OR "young person*" OR "young people" OR AYA OR TYA OR adolescen* OR teen* OR youngster* OR pre-teen)
**Block 3: Cancer**	(neoplasm* OR cancer* OR carcinoma* OR tumor* OR tumour* OR malignan* OR leukemia* OR leukaemia* OR sarcoma* OR radiotherapy OR chemotherapy)
**Block 4: Survivorship**	(Survivor OR survivors OR Long-Term Survivors OR Long Term Survivors OR Long-Term Survivor OR Survivor, Long-Term OR Survivors, Long-Term OR survivo* OR survivi*)
**Search**	Block 1 AND Block 2 AND Block 3 AND Block 4

Table S5: Quality assessment for quantitative cross-sectional studies.

1. Were the criteria for inclusion in the sample clearly defined?
2. Were the study subjects and the setting described in detail?
3. Was the exposure measured in a valid and reliable way?
4. Were objective, standard criteria used for measurement of the condition?
5. Were confounding factors identified?
6. Were strategies to deal with confounding factors stated?
7. Were the outcomes measured in a valid and reliable way?
8. Was appropriate statistical analysis used?

Table S6: Quality assessment for qualitative studies.

1. Is there congruity between the stated philosophical perspective and the research methodology?
2. Is there congruity between the research methodology and the research question or objectives?
3. Is there congruity between the research methodology and the methods used to collect data?
4. Is there congruity between the research methodology and the representation and analysis of data?
5. Is there congruity between the research methodology and the interpretation of results?
6. Is there a statement locating the researcher culturally or theoretically?
7. Is the influence of the researcher on the research, and vice-versa, addressed?
8. Are participants, and their voices, adequately represented?
9. Is the research ethical according to current criteria or, for recent studies, and is there evidence of ethical approval by an appropriate body?
10. Do the conclusions drawn in the research report flow from the analysis, or interpretation, of the data?
